# Integrated multi-omics and machine learning reveals immune-metabolic signatures in osteoarthritis: from bulk RNA-seq to single-cell resolution

**DOI:** 10.3389/fimmu.2025.1599930

**Published:** 2025-06-16

**Authors:** Hui He, Xiumei Zhao, Bo Zhang, Shijian Zhao, Yinteng Wu

**Affiliations:** ^1^ Department of Orthopedic and Trauma Surgery, The Third Affiliated Hospital of Guangxi Medical University, Nanning, Guangxi, China; ^2^ School of Clinical Medicine, Youjiang Medical University for Nationalities, Baise, Guangxi, China; ^3^ Graduate School, Kunming Medical University, Kunming, Yunnan, China

**Keywords:** osteoarthritis (OA), immune-metabolism, weighted gene co-expression network analysis (WGCNA), machine learning, genome-wide association studies

## Abstract

**Purpose:**

The aim of this study was to investigate the activation of immune-metabolic pathways in osteoarthritis (OA) and their role in disease progression. We employed differential expression analysis and Gene Set Enrichment Analysis

**Materials and methods:**

Gene set enrichment analysis (GSEA) to identify activated immune-metabolism pathways in OA. Subsequently, Weighted gene co-expression network analysis (WGCNA) was used to identify gene modules associated with OA and immune-metabolism scores, followed by enrichment analysis to reveal the functional characteristics of these modules. To identify immune-metabolism related differentially expressed genes (DEGs), we utilized seven machine learning methods, including lasso regression, random forest, bagging, gradient boosting machines (GBM), Xgboost-xgbLinear, Xgboost-xgbtree, and decision trees, to construct predictive models and validate their reliability. Based on the expression profiles of hub immune-metabolism related DEGs, we stratified OA patients into two immune-metabolism related subgroups and deeply investigated the differences in immune profiles, drug responses, functions, and pathways between these subgroups. Additionally, we analyzed the expression and pseudotime trajectories of hub immune-metabolism related DEGs at the single-cell level. Through genome-wide association studies (GWAS), we explored the mechanisms of action of hub immune-metabolism related DEGs. Finally, real-time polymerase chain reaction (RT-PCR) was utilized to verify the expression of hub immune-metabolism related DEGs.

**Results:**

Immune-metabolism related pathways were significantly activated during the development of OA. Thirteen central immune metabolism-related genes (CX3CR1, ADIPOQ, IL17RA, APOD, EGFR, SPP1, PLA2G2A, CXCL14, RARB, ADM, CX3CL1, TNFSF10, and MPO) were identified. Predictive modeling by constructing these genes has good predictive power for identifying OA. These genes are mainly associated with endothelial cells. Single-cell analysis showed that they were all expressed in single cells and varied with cell differentiation. RT-PCR results suggested that they were all significantly expressed in OA.

**Conclusion:**

Our findings indicate that immune metabolism plays a key role in the development of OA and provide new perspectives for future therapeutic strategies

## Introduction

Osteoarthritis (OA) is a chronic, multifaceted joint disease characterized primarily by the progressive degradation of articular cartilage. This condition can lead to significant joint dysfunction and persistent pain (1). Statistics indicate that over 500 million individuals worldwide are affected by osteoarthritis, making it a leading cause of disability among the elderly population (2). Currently, there is no definitive cure for OA, partly due to a lack of comprehensive understanding regarding the pathological mechanisms underlying its onset and progression. Therefore, elucidating the critical pathological signaling pathways and key molecular players involved in the pathogenesis of OA is essential for the design of targeted therapies and the development of novel pharmacological interventions.

Currently, many of humanity’s persistent ailments are closely linked to dysregulation of the immune system. The immune system is tightly regulated by metabolic processes, which can influence disease progression through alterations in metabolism (3). Immunometabolism has emerged as a significant and burgeoning field of study. Cellular metabolism plays a crucial role in guiding and modulating the differentiation and functionality of immune cells. It is not only a fundamental process that sustains cellular life but also serves as a critical determinant of cellular adaptability (4). Recent research indicates that metabolism plays a pivotal role in inflammatory arthritic diseases. In particular, metabolic alterations and aberrant immunometabolism may represent key characteristics of the various phenotypes of OA (5). Additionally, metabolism is vital for the functionality of cartilage and synovial joints. Under pathological conditions, cells transition from a quiescent state to a highly metabolically active state to maintain energy homeostasis. This phenomenon also leads to the production of inflammatory and catabolic proteins, which subsequently activate essential transcription factors and increase the biosynthetic metabolites involved in inflammatory signaling pathways, thereby propelling the ongoing progression of the disease. Abnormal chondrocyte metabolism is a response to changes in the immune microenvironment and may play a significant role in cartilage degradation and the progression of OA (6). Therefore, alterations in the immune microenvironment and changes in chondrocyte metabolism may serve as distinctive features of the different OA phenotypes. Future research should place greater emphasis on the interplay between immune and metabolic pathways to enhance our understanding of the pathophysiology of OA. Such insights will be instrumental in identifying therapeutic targets associated with OA and metabolic pathways, potentially facilitating the development of novel pharmacological agents for the treatment of this condition.

This study employed differential expression analysis and gene set enrichment analysis (GSEA) to investigate the activation of immune-metabolic pathways in OA. Subsequently, we applied weighted gene co-expression network analysis (WGCNA) to identify gene modules associated with OA and immune-metabolic scores, followed by enrichment analyses to elucidate the functional characteristics of these modules. To identify differentially expressed genes (DEGs) associated with immuno-metabolism, we used seven machine learning methods including lasso regression, random forest, Bagging, GBM, Xgboost-xgbLinear, Xgboost-xgbtree, and Decision Tree, and constructed the prediction models and verified their reliability. Based on the expression profiles of hub immune-metabolic related DEGs, we classified OA patients into two distinct immune-metabolic subgroups and conducted an in-depth exploration of the differences between these subgroups in terms of immune landscape, drug response, functionality, and pathway involvement. Additionally, we analyzed the expression and pseudotemporal dynamics of hub immune-metabolic related DEGs at the single-cell level. Finally, through genome-wide association studies (GWAS), we investigated the mechanisms underlying the role of hub immune-metabolic related DEGs.

## Materials and methods

### Data acquisition and preprocessing

In this study, we initially downloaded osteoarthritis-related microarray datasets from the Gene Expression Omnibus (GEO) database (https://www.ncbi.nlm.nih.gov/geo/), including GSE117999, GSE12021, GSE51588, GSE55235, GSE55457, GSE57218, GSE82107, and GSE98918. Additionally, we retrieved single-cell sequencing data related to osteoarthritis from the GEO database under the accession number GSE104782. During the processing of the microarray datasets, we matched probes to gene names based on the annotation information for each GPL platform, prioritizing the probes with the highest expression levels to ensure accuracy and consistency. Subsequently, we employed the “normalizeBetweenArrays” function to standardize the expression matrix and processed the datasets requiring log2 transformation. We then compiled a list of common genes across these eight datasets to serve as the foundation for our subsequent analyses. To address the discrepancies in expression values stemming from different batches or platforms, we utilized the ComBat method from the “sva” package (7) in R for normalization. Principal component analysis (PCA) was conducted to evaluate the success of batch effect removal, thereby ensuring the reliability of the data. We obtained immune-related genes from the Immport database (https://www.immport.org/) and metabolic-related genes from the Harmonizome database (https://maayanlab.cloud/Harmonizome/). By intersecting these two gene sets, we identified immune-metabolic related genes, which were subsequently utilized for further analyses.

### Differential expression analysis

DEGs between OA samples and normal samples was conducted using the “limma” R package (8), specifically focusing on the identification of immune-metabolic related DEGs. The selection criterion for significant DEGs was set at a p-value threshold of <0.05. To visualize the differential expression of immune-metabolic related DEGs, we employed volcano plots, which effectively illustrate the significance and magnitude of changes in gene expression. Additionally, heatmaps were utilized to display the expression levels of these DEGs across each sample, providing a clear comparative overview. Furthermore, we analyzed the correlation among immune-metabolic related DEGs and employed the “wilcox.test” algorithm to assess their differential expression levels between OA and normal samples. This comprehensive analysis allowed us to elucidate the specific contributions of immune-metabolic pathways in the context of OA.

### Enrichment analysis

Initially, immune-metabolic related DEGs were compiled into a gene set, which was then subjected to GSEA using the “clusterProfiler” R package (9) to explore their activation or inhibition status in the context of OA. This analysis aimed to elucidate the functional implications of these genes within the disease milieu. Additionally, we employed the “clusterProfiler” R package to conduct gene ontology (GO) (10) and kyoto encyclopedia of genes and genomes (KEGG) (11) analyses on the immune-metabolic related DEGs. This approach allowed us to uncover the functional characteristics and pathway information associated with these genes, providing deeper insights into their roles in OA pathology and the underlying immune-metabolic interactions.

### Enrichment landscape of immune-metabolic related DEGs in immune and metabolic pathway

We first analyzed the upregulation or downregulation of immune-related and metabolic pathways in OA samples compared to control samples. Following this, we employed the single-sample gene set enrichment analysis (ssGSEA) method to calculate the activity scores for each pathway. To further elucidate the relationship between immune-metabolic related DEGs and these pathways, we utilized the Spearman correlation algorithm to assess the correlation between each DEG and the respective pathway activity scores. This comprehensive analysis provided insights into the involvement of immune-metabolic related DEGs in specific immune and metabolic signaling pathways within the OA context.

### WGCNA analysis

In this study, immune-metabolic related DEGs were utilized as the background gene set. We calculated the gene set variation analysis (GSVA) scores for each sample and used these scores as traits for the WGCNA (12). After selecting the top 5000 highly variable genes, we determined the soft thresholding power for the scale-free network to achieve the maximum R² value (power = 4). To ensure that each module contained a sufficient number of genes, we set a minimum requirement of at least 30 genes per module. We assessed the distances between genes using the topology overlap matrix similarity. Hierarchical clustering analysis was performed using both average linkage and dynamic tree cut methods to construct a clustering dendrogram, thereby classifying genes into distinct modules and merging them based on their similarities. Subsequently, we selected the modules most strongly correlated with the GSVA scores for enrichment analysis. This analysis was conducted using the Metascape database for GO and KEGG pathway analyses, providing insights into the functional roles of the identified gene modules within the context of osteoarthritis.

### Identification of hub immune-metabolic related DEGs

To identify hub immune-metabolic related DEGs, this study employed the Lasso algorithm as a primary method. Additionally, we utilized several other machine learning techniques, including Random Forest, Bagging, Gradient Boosting Machine (GBM), Xgboost (both xgbLinear and xgbtree variants), and Decision Tree methods to rank the importance of genes. These combined approaches enabled us to filter and prioritize the top 40 most significant genes based on their importance scores. The consensus from these methods established robust criteria for the identification of hub immune-metabolic related DEGs, facilitating further analysis and insights into their roles within OA.

### Development and evaluation of machine learning models

To identify the optimal machine learning model for predicting OA, we selected input variables from the expression profiles of 13 immune-metabolic related genes and their corresponding grouping information. Utilizing the “mlr3verse” R package, we established seven different machine learning models, including Logistic Regression, Support Vector Machine (SVM), k-Nearest Neighbors (kknn), Random Forest, Linear Discriminant Analysis (LDA), Naive Bayes, Decision Tree. To validate the reliability of these models, we performed a model performance evaluation using the GSE48556 validation dataset. In addition to the above models, we also constructed a Convolutional Neural Network (CNN) using the “keras” R package. The performance of this CNN model was similarly assessed using the GSE48556 validation set, allowing us to compare its predictive capabilities against the traditional machine learning models.

### Construction and validation of the nomogram

This study employed multivariate logistic regression analysis to evaluate 13 immune-metabolic related genes. The “ROCR” package was utilized to compute the area under the receiver operating characteristic curve (AUC), thereby assessing the diagnostic value of these genes in OA. We constructed a nomogram to predict the probability of OA occurrence and generated calibration curves and decision curves to analyze the stability and reliability of the model.

### Non-negative matrix factorization algorithm

Based on the expression profile data of immune-metabolic genes, we employed the Non-negative Matrix Factorization (NMF) algorithm to decompose the matrix for OA samples, resulting in a coefficient matrix for each sample and a contribution matrix for each gene set. These matrices elucidate the relationships between the samples and gene sets. Utilizing clustering algorithms, we assigned the samples to distinct clusters and provided annotations for each cluster. In selecting the optimal value of (k), we assessed various metrics including cophenetic correlation, dispersion, and silhouette scores. Ultimately, using the aforementioned algorithms along with the optimal (k) value, we classified OA samples into different molecular clusters.

### Characterization of subtype features

We employed ssGSEA to assess the activity levels of Subtype 1 and Subtype 2. Additionally, we analyzed the differential expression levels of hub immune-metabolic related DEGs and immune cells between these subtypes. Variations in immune and metabolic pathways within the subtypes were also examined. To identify potential therapeutic agents for patients with Subtype 1 and Subtype 2, we utilized the Connectivity Map (cMAP) database. Furthermore, we conducted protein annotation and functional analysis for Subtype 1 and Subtype 2 using the ProteoMap database. Finally, GSVA was performed to identify differential pathways between Subtype 1 and Subtype 2.

### WGCNA for subtype analysis

Based on the characteristics of the subtypes, we employed WGCNA to identify potential functional modules that characterize the biological functions of each subtype. We selected the top 5000 highly variable genes and determined the optimal soft threshold for the scale-free network to achieve the maximum (R^2) value (power = 4). Each module was required to contain a minimum of 30 genes. We assessed the distances among gene pairs using the topology overlap matrix similarity. Subsequently, we conducted hierarchical clustering analysis using both average linkage and dynamic tree cutting methods to construct a clustering dendrogram, thereby categorizing the genes into distinct modules. For the functional analysis of key modules, we utilized the “ClusterProfiler” R package to perform GO and KEGG analyses.

### Single-cell analysis

We processed single-cell RNA sequencing data using the “Seurat” R package. Cells expressing more than 200 genes but fewer than 2500 genes were identified for further analysis. High-variance genes were detected using the “FindVariableGenes” function, followed by principal component analysis (PCA). For dimensionality reduction and visualization of single-cell data, we employed the Uniform Manifold Approximation and Projection (UMAP) method. Cell types were annotated. We visualized the clustering of cells using the “DimPlot” function and illustrated gene expression patterns with the “FeaturePlot” function.

### Pseudotime analysis

A subset of endothelial cells (ECs) was extracted for pseudotime analysis. We reprocessed the chondrocytes for dimensionality reduction and clustering. The “Monocle” R package was utilized to conduct the pseudotime analysis. For subsequent pseudotime analysis, we selected cells based on the criteria of mean expression greater than 0.1 and empirical dispersion greater than 1 times the fitted dispersion. Dimensionality reduction was performed using the “reduceDimension” function with the “DDRTree” method, followed by trajectory ordering. The “plot_cell_trajectory” function was employed to visualize the distribution of cells along the trajectory. Additionally, we analyzed the expression changes of hub immune-metabolic related genes among different clusters throughout the cellular differentiation trajectory.

### Cell communication analysis

A subset of ECs was extracted, and based on the immune-metabolic gene set scoring, we defined chondrocytes with scores greater than the fourth quartile as high immune-metabolic scoring ECs. Conversely, ECs with scores below the fourth quartile were classified as low immune-metabolic scoring chondrocytes. Cell communication analysis was conducted using the “CellChat” R package.

### GWAS analysis

The Gene Atlas database (http://geneatlas.roslin.ed.ac.uk) is a comprehensive resource that provides extensive information on associations between hundreds of traits and millions of variants, utilizing data from the UK Biobank cohort. This database encompasses data from 452,264 individuals in the UK Biobank, covering a wide range of 778 phenotypes and 30 million genetic loci.

### RT-PCR validation

Human chondrocytes (Wuhan Saos Technology Co., Ltd.) were cultured in DMEM/F12 medium containing 10% fetal bovine serum. To model inflammation, cells in the intervention group were exposed to interleukin-1β (IL-1β; 10 ng/ml) for 24 hours. Total RNA was isolated using QIAzol reagent, reverse-transcribed into cDNA with oligo-dT primers, and amplified by quantitative reverse-transcription polymerase chain reaction (qRT-PCR) under the following conditions: initial denaturation at 95°C for 5 minutes; 40 cycles of denaturation (95°C, 1 minute), annealing (60°C, 30 seconds), and extension (72°C, 1 minute). Gene expression was quantified via the 2−ΔΔCt method. Primer sequences are detailed in **Table 1**.

**Table 1:** Primers used in this study.

Primer	Sequence
CX3CR1-F	ACTTTGAGTACGATGATTTGGCT
CX3CR1-R	GGTAAATGTCGGTGACACTCTT
IL17RA-F	AGTTCCACCAGCGATCCAAC
IL17RA-R	GTCTGAGGCAGTCATTGAGGC
APOD-F	GAATCAAATCGAAGGTGAAGCCA
APOD-R	ACACGAGGGCATAGTTCTCAT
EGFR-F	AGGCACGAGTAACAAGCTCAC
EGFR-R	ATGAGGACATAACCAGCCACC
SPP1-F	CTCCATTGACTCGAACGACTC
SPP1-R	CAGGTCTGCGAAACTTCTTAGAT
PLA2G2A-F	ATGAAGACCCTCCTACTGTTGG
PLA2G2A-R	GCTTCCTTTCCTGTCGTCAACT
RARB-F	TCCGAAAAGCTCACCAGGAAA
RARB-R	GGCCAGTTCACTGAATTTGTCC
ADM-F	ATGAAGCTGGTTTCCGTCG
ADM-R	GACATCCGCAGTTCCCTCTT
ADIPOQ-F	TGCTGGGAGCTGTTCTACTG
ADIPOQ-R	TACTCCGGTTTCACCGATGTC
CX3CL1-F	GCCACAGGCGAAAGCAGTA
CX3CL1-R	GGAGGCACTCGGAAAAGCTC
TNFSF10-F	TGCGTGCTGATCGTGATCTTC
TNFSF10-R	GCTCGTTGGTAAAGTACACGTA
MPO-F	CCAGATCATCACTTACCGGGA
MPO-R	CACTGAGTCATTGTAGGAACGG
CXCL14-F	CGCTACAGCGACGTGAAGAA
CXCL14-R	GTTCCAGGCGTTGTACCAC
GAPDH-F	TCAAGATCATCAGCAATGCC
GAPDH-R	CGATACCAAAGTTGTCATGGA

## Results

### Identification and enrichment analysis of immune-metabolic related DEGs

**Figure 1:**
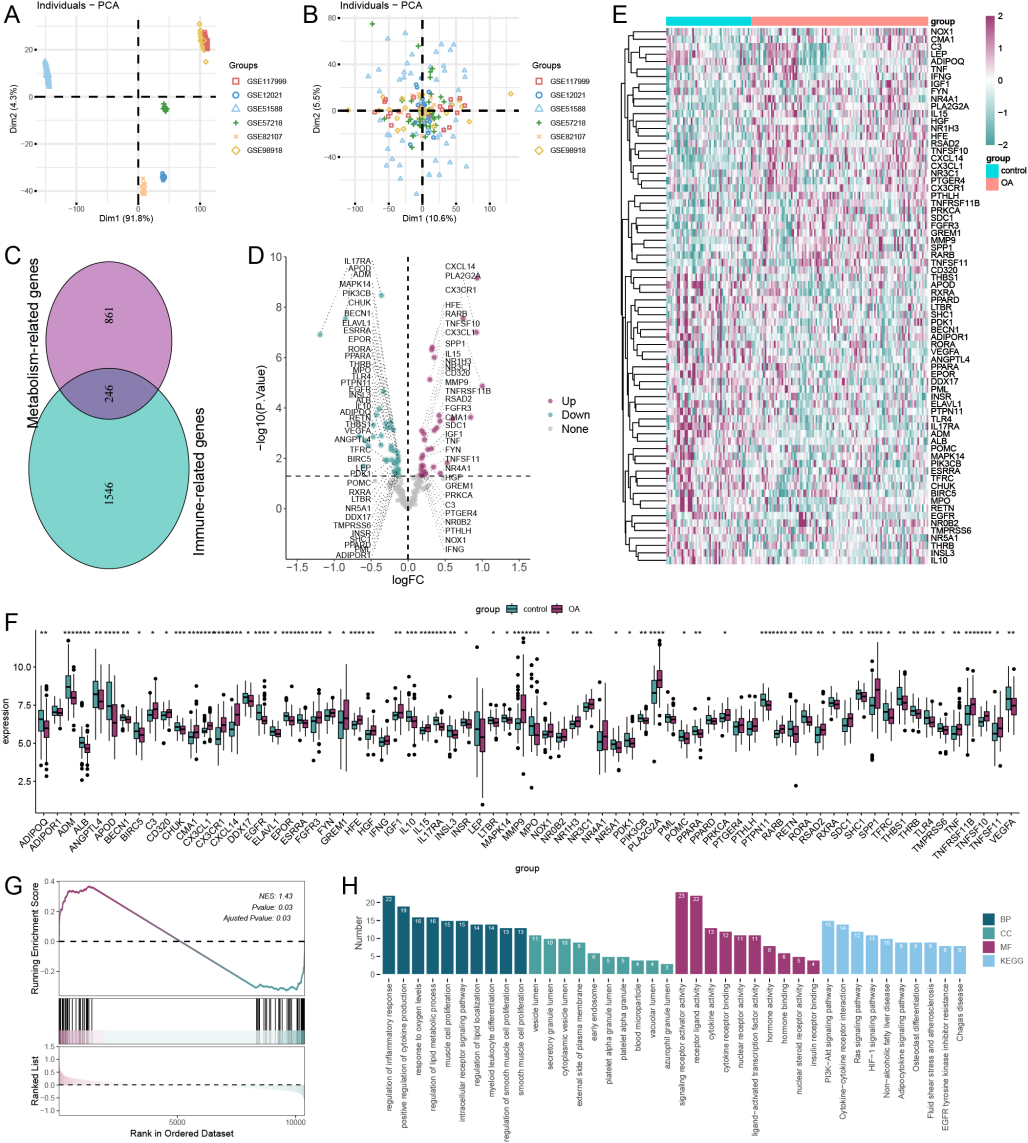
Data preprocessing and enrichment analysis. principal component analysis of six dataset batches before **(A)** and after **(B)** correction. **(C)** Wayne plot showing common intersecting genes of immune-related and metabolism-related genes. **(D)** Volcano plot showing the results of differential expression analysis of immune-metabolism-related genes. **(E)** Heatmap of immune-metabolism-related genes. **(F)** wilcox.test analysis of expression levels of immune-metabolism-related genes (*p < 0.05, **p < 0.01, ***p < 0.001, ****p < 0.0001). **(G)** GSEA showed that the immune-metabolism-related gene set was significantly activated in OA. **(H)** Results of GO and KEGG enrichment analysis of immune-metabolic related genes. GO, Gene Ontology; BP, Biological Process; CC, Cellular Component; MF, Molecular Function; KEGG, Kyoto Encyclopedia of Genes and Genomes.

As illustrated in **Figure 1A**, samples from eight independent datasets exhibited varying batch effects; however, following the removal of these effects, the samples clustered together (**Figure 1B**). This indicates that cross-platform normalization successfully mitigated batch processing effects, allowing for subsequent analyses. We identified a total of 246 immune-metabolic genes using data from the Harmonizome database and the ImmPort database (**Figure 1C**). The differential expression analysis revealed a total of 67 Immune-metabolic related DEGs, comprising 31 upregulated and 36 downregulated genes (**Figure 1D**). A heatmap displayed the expression levels of these Immune-metabolic related DEGs across each sample (**Figure 1E**). Furthermore, rank sum test analysis confirmed that the Immune-metabolic related DEGs exhibited statistically significant expression differences (**Figure 1F**). GSEA indicated that the immune-metabolic gene set was significantly activated in OA (**Figure 1G**). Enrichment analysis demonstrated that the Immune-metabolic related DEGs were notably enriched in the following pathways: regulation of inflammatory response, positive regulation of cytokine production, regulation of lipid metabolic processes, PI3K-Akt signaling pathway, and cytokine-cytokine receptor interaction (**Figure 1H**).

### Correlation analysis of immune-metabolic related DEGs with immune and metabolic pathways

**Figure 2:**
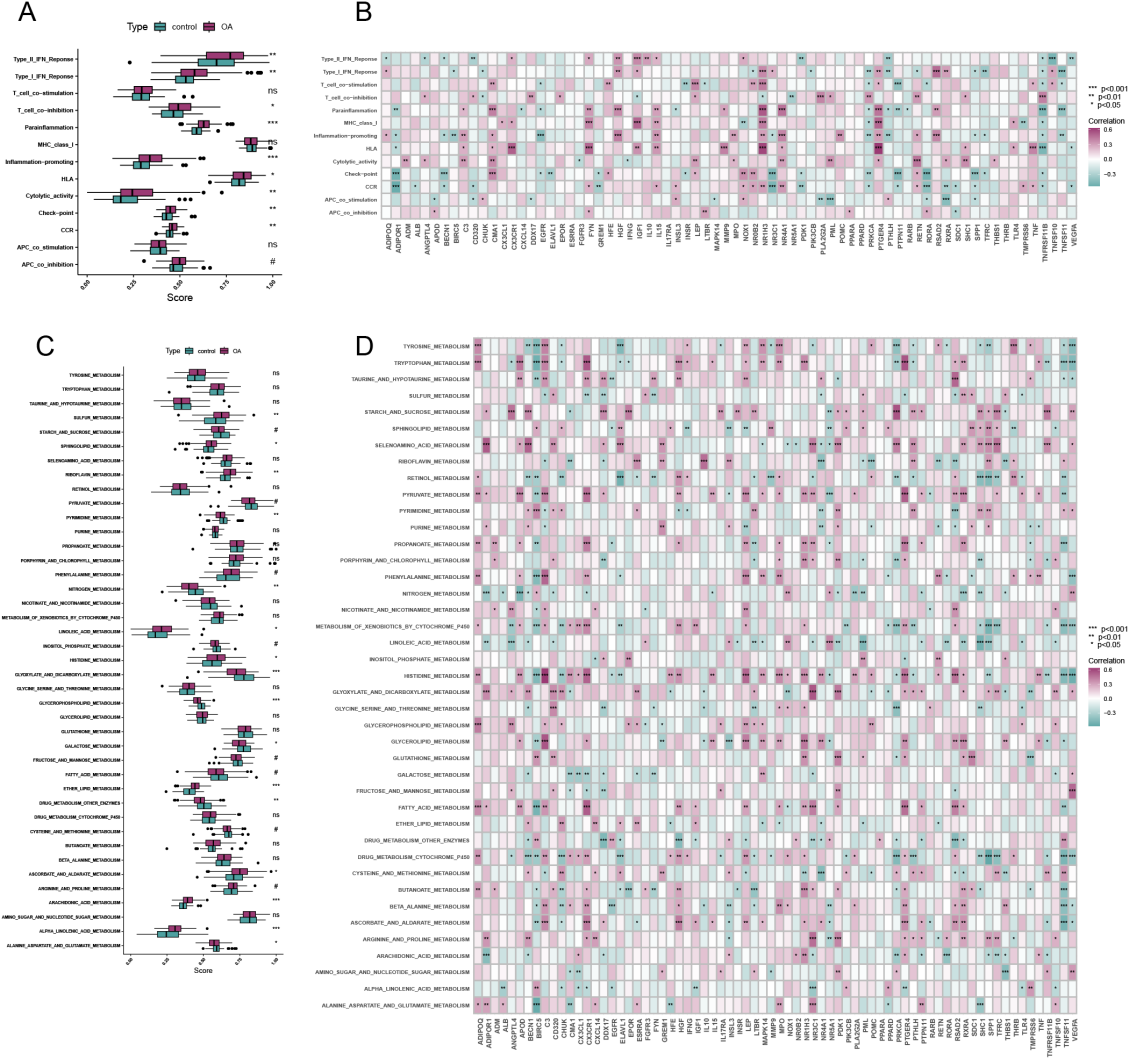
Pathway correlation analysis. **(A)** Differential analysis of immune-related pathways in OA and control. **(B)** Correlation analysis of immune-metabolism-related differentially expressed genes and immune-related pathways. **(C)** Differential analysis of metabolism-related pathways in OA and control. **(D)** Correlation analysis of immune-metabolic related differentially expressed genes and metabolism-related pathways. (*p < 0.05, **p < 0.01, ***p < 0.001, ****p < 0.0001).

**Figures 2A, C** illustrate a significant increase in the activity of most immune-related pathways and metabolic pathways in OA. **Figures 2B, D** present the results of the correlation analysis between immune-metabolic related genes and both immune-related and metabolic pathways, respectively. These analyses underscore the interconnectedness of differential gene expression and pathway activation in the context of OA.

### Construction of co-expression network

**Figure 3:**
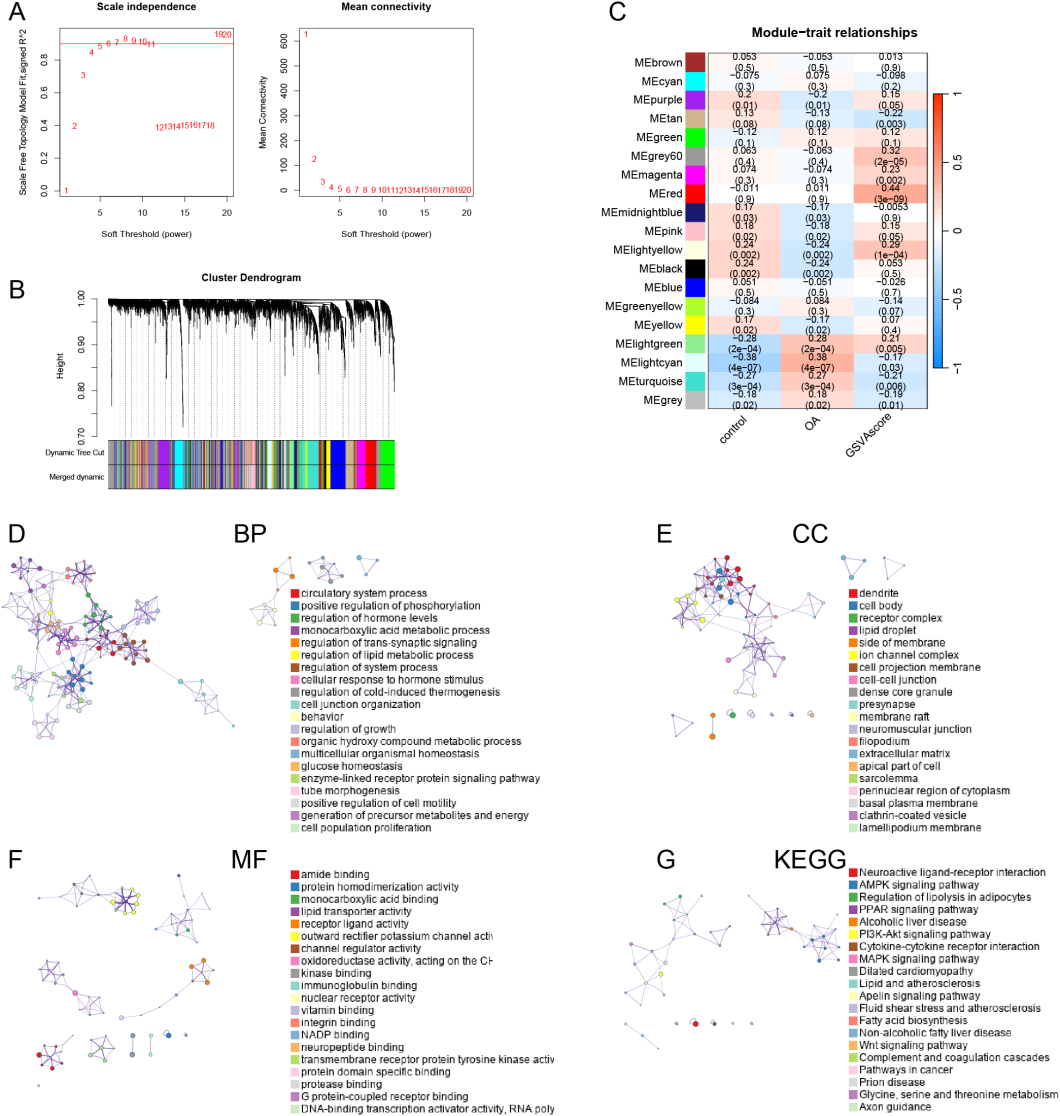
Weighted Gene Co-expression Network Analysis. **(A)** the dendrogram of gene clustering based on topological overlapping. **(B)** Optimal soft threshold screening (The vertical axis of the left panel represents the square of the correlation coefficient between log(k) and log(p(k)) in the corresponding network, the higher the square of the correlation coefficient, the closer the network is to the distribution without network scales; the vertical axis of the right panel represents the mean value of the neighbor-joining function of all genes in the corresponding gene module). **(C)** Correlation analysis of modules and traits. Enrichment analyses of the red module genes, including BP **(D)**, CC **(E)**, MF **(F)** and KEGG **(G)**.

We established an optimal soft threshold of 5 to construct a scale-free network (**Figure 3A**). Using the optimal dynamic tree cut method and hierarchical clustering, we merged similar modules (**Figure 3B**). Correlation analysis between modules and traits revealed that the red module had the strongest association with the immune-metabolic GSVA score (**Figure 3C**). Subsequently, we performed enrichment analysis on the genes within the red module using the “Metascape” database. In terms of Biological Processes (BP), the genes in the red module were significantly enriched in circulatory system processes, monocarboxylic acid metabolic processes, regulation of lipid metabolic processes, and organic hydroxy compound metabolic processes (**Figure 3D**). For Cellular Components (CC), the red module genes were notably enriched in dendrites, lipid droplets, and cell bodies (**Figure 3E**). In terms of Molecular Functions (MF), these genes showed significant enrichment in amide binding, protein homodimerization activity, and monocarboxylic acid binding (**Figure 3F**). Lastly, in the KEGG pathway analysis, the red module genes were significantly enriched in neuroactive ligand-receptor interaction, AMPK signaling pathway, and regulation of lipolysis in adipocytes (**Figure 3G**).

### Screening of immune-metabolic related hub genes

**Figure 4:**
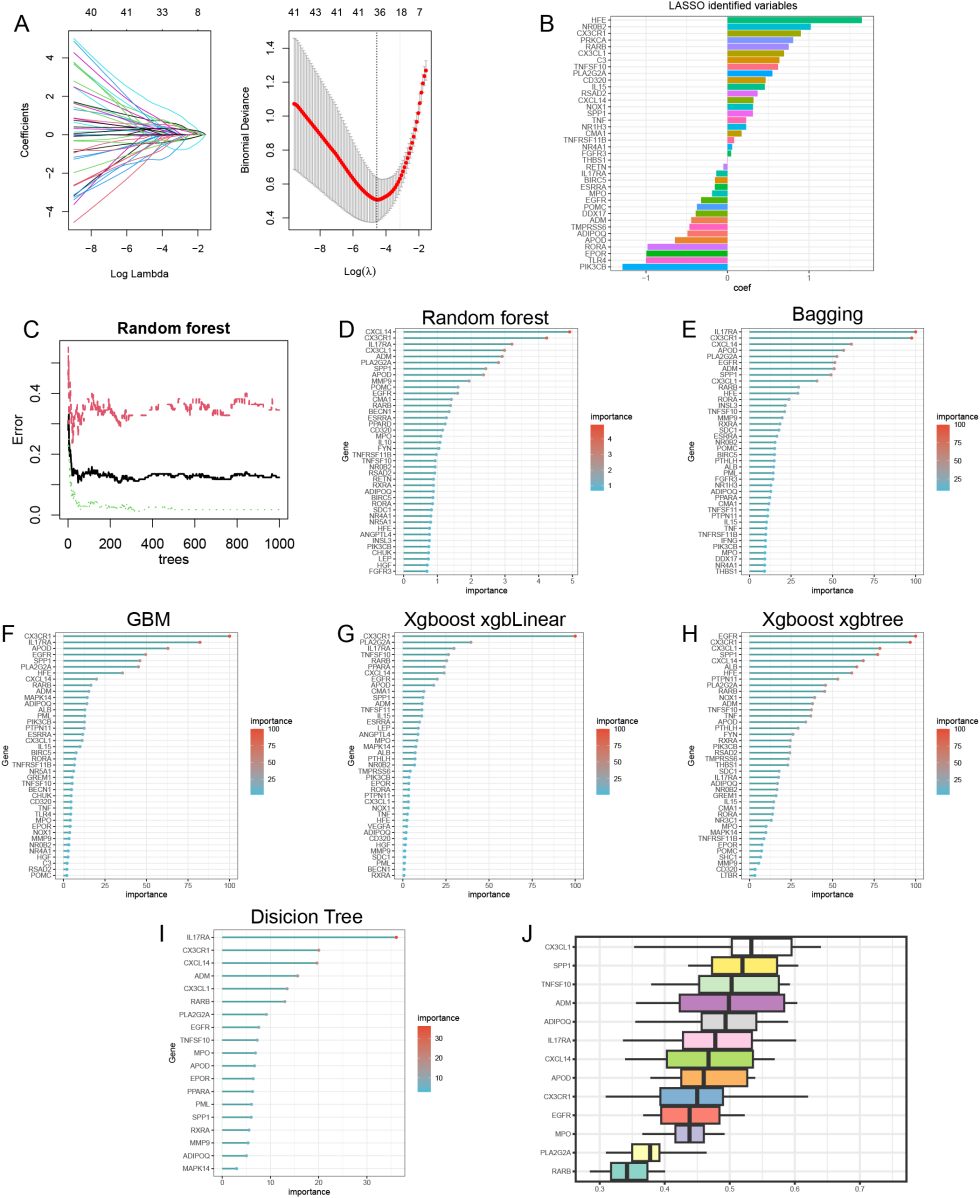
Machine learning identification of immune-metabolism related hub genes. **(A)** The lasso analysis identified 38 immune-metabolism related genes. Log (Lambda) value of the seven genes in the lasso model (left panel) and the most proper log (Lambda) value in the lasso model (right panel). **(B)** The coef values of the 38 immune-metabolism related genes in the lasso model. **(C)** The random forest model has the lowest error rate when XX trees are selected. Random forest **(D)**, bagging **(E)**, GBM **(F)**, Xgboost-xgbLinear **(G)** and Xgboost-xgbtree **(H)** identified top40 important immune-metabolic related genes. decision tree identified 19 important immune-metabolic related genes **(I)**. results of Friends analysis **(J)**.

Lasso analysis identified a total of 38 immune-metabolic related genes (**Figure 4A**). **Figure 4B** shows the coefficient values (coef) of these 38 genes. Subsequently, we employed several machine learning approaches—including Random Forest (**Figures 4C-D**), Bagging (**Figure 4E**), Gradient Boosting Machine (GBM) (**Figure 4F**), XGBoost with xgbLinear (**Figure 4G**), and XGBoost with xgbtree (**Figure 4H**)—to identify the top 40 important immune-metabolic related genes. Additionally, a decision tree analysis revealed 19 significant immune-metabolic related genes (**Figure 4I**). Through the integration of results from these seven algorithms, we collectively identified 13 immune-metabolic related hub genes (**Supplementary Figure 2**). Furthermore, Friends analysis indicated that these 13 hub genes exhibit functional similarities (**Figure 4J**).

### Establishment and validation of machine learning models and convolutional neural networks

**Figure 5:**
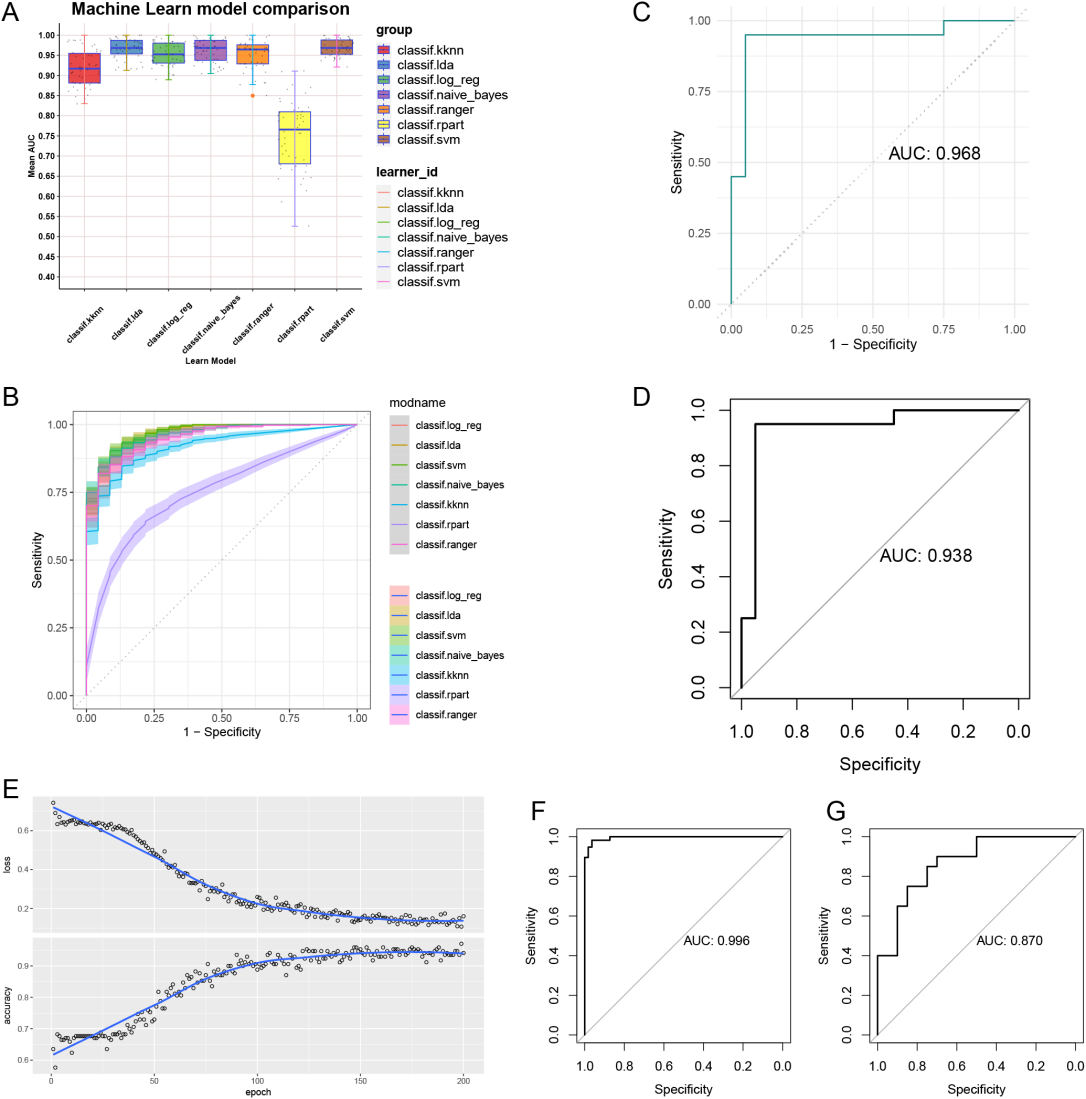
Machine learning models and convolutional neural networks built and validated. **(A)** Comparison of 7 machine learning models AUC. **(B)** Comparison of ROC of 7 machine learning models. **(C)** AUC of SVM model. **(D)** AUC of validation set. **(E)** Accuracy and loss assessment of convolutional neural network. AUC of convolutional neural networks for training set **(F)** and validation set **(G)**.

By comparing seven different machine learning models, we found that the SVM model exhibited the highest AUC value (**Figure 5A**). Additionally, the SVM model demonstrated good sensitivity and specificity (**Figure 5B**). The AUC for the SVM model was 0.968 (**Figure 5C**), indicating its strong ability to identify patients with OA. The AUC value for the validation set was 0.938, further confirming the reliability of the model (**Figure 5D**). In constructing the CNN, we observed significant improvements in model accuracy from the initial step to the final step (**Figure 5E**). The AUC for the training set reached 0.996 (**Figure 5F**), while the AUC for the validation set was 0.87 (**Figure 5G**).

### Nomogram model for risk prediction

**Figure 6:**
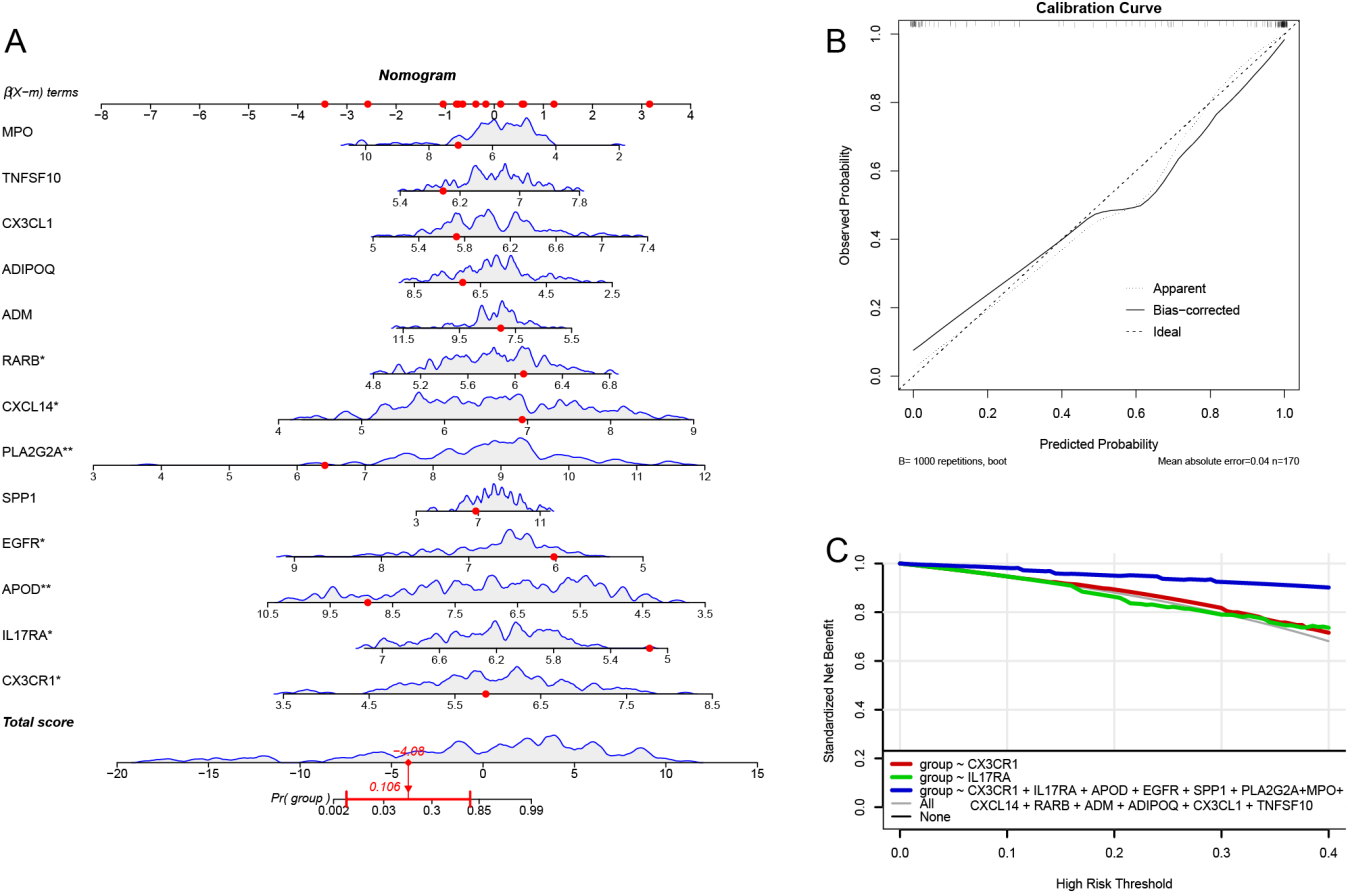
Column-line diagram model. **(A)** Constructing the column-line diagram. **(B)** Calibration curve for the diagnostic model. **(C)** Model evaluation curves.

We constructed a risk prediction nomogram model (**Figure 6A**). To internally validate this nomogram model, we employed the Bootstrap method with 1000 resampling iterations. The results indicated that the calibration curve of the model closely approached the ideal line, suggesting a high level of reliability in the predictions (**Figure 6B**). Additionally, DCA demonstrated that the model possesses good predictive ability and clinical utility (**Figure 6C**).

### NMF analysis

**Figure 7:**
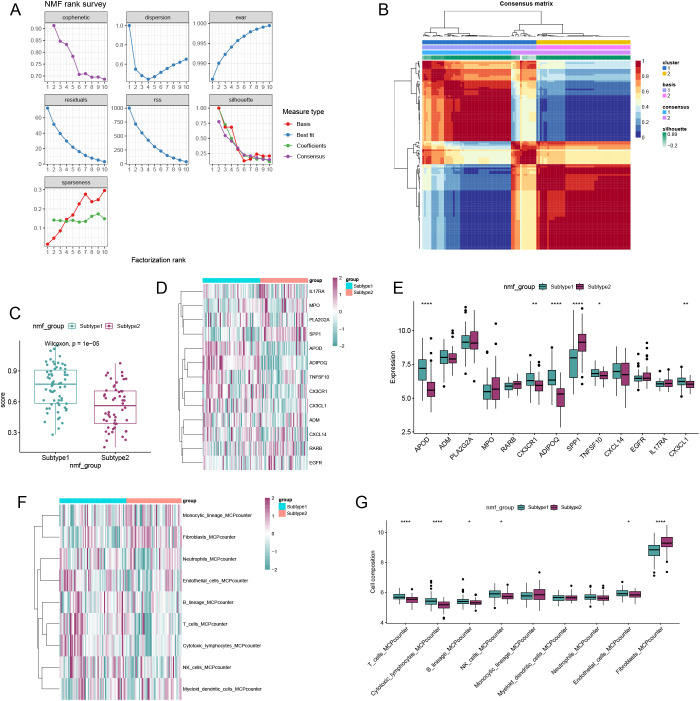
Non-negative matrix factorization (NMF) analysis for the OA samples. **(A)** Distribution of cophenetic, residuals, RSS and silhouette with a rank of 2–10. **(B)** Consensus map of NMF clustering when k = 2. **(C)** ssGSEA algorithm showed that subgroup1 and subgroup2 had higher activity. **(D)** Heatmap showing the expression landscape of DE-SMRGs of the two clusters. **(E)** Box plot showing the expression of 13 DE-SMRGs between the two clusters. **(F)** Heatmap showing the expression landscape of 9 kinds of immune cells of the two clusters. **(G)** Box plot showing the expression of 9 kinds of immune cells between the two clusters (*p < 0.05, **p < 0.01, ***p < 0.001, ****p < 0.0001).

We employed the Non-negative Matrix Factorization (NMF) algorithm to perform clustering analysis based on the expression profiles of 13 immune-metabolic related hub genes across all OA samples. Comprehensive evaluation using cophenetic, dispersion, and silhouette metrics led us to determine that (k=2) was the optimal number of clusters (**Figures 7A, B**). Consequently, we classified all OA samples into two distinct clusters according to the NMF algorithm. Utilizing the single-sample Gene Set Enrichment Analysis (ssGSEA) algorithm, we found that subcluster 1 exhibited higher activity compared to subcluster 2 (**Figure 7C**). Most immune-metabolic related hub genes were highly expressed in subcluster 1 (**Figures 7D-E**). Furthermore, six types of immune cells showed increased expression levels in subcluster 1 (**Figures 7F-G**).

### Immune activity, metabolic activity, and drug analysis of subtypes

**Figure 8:**
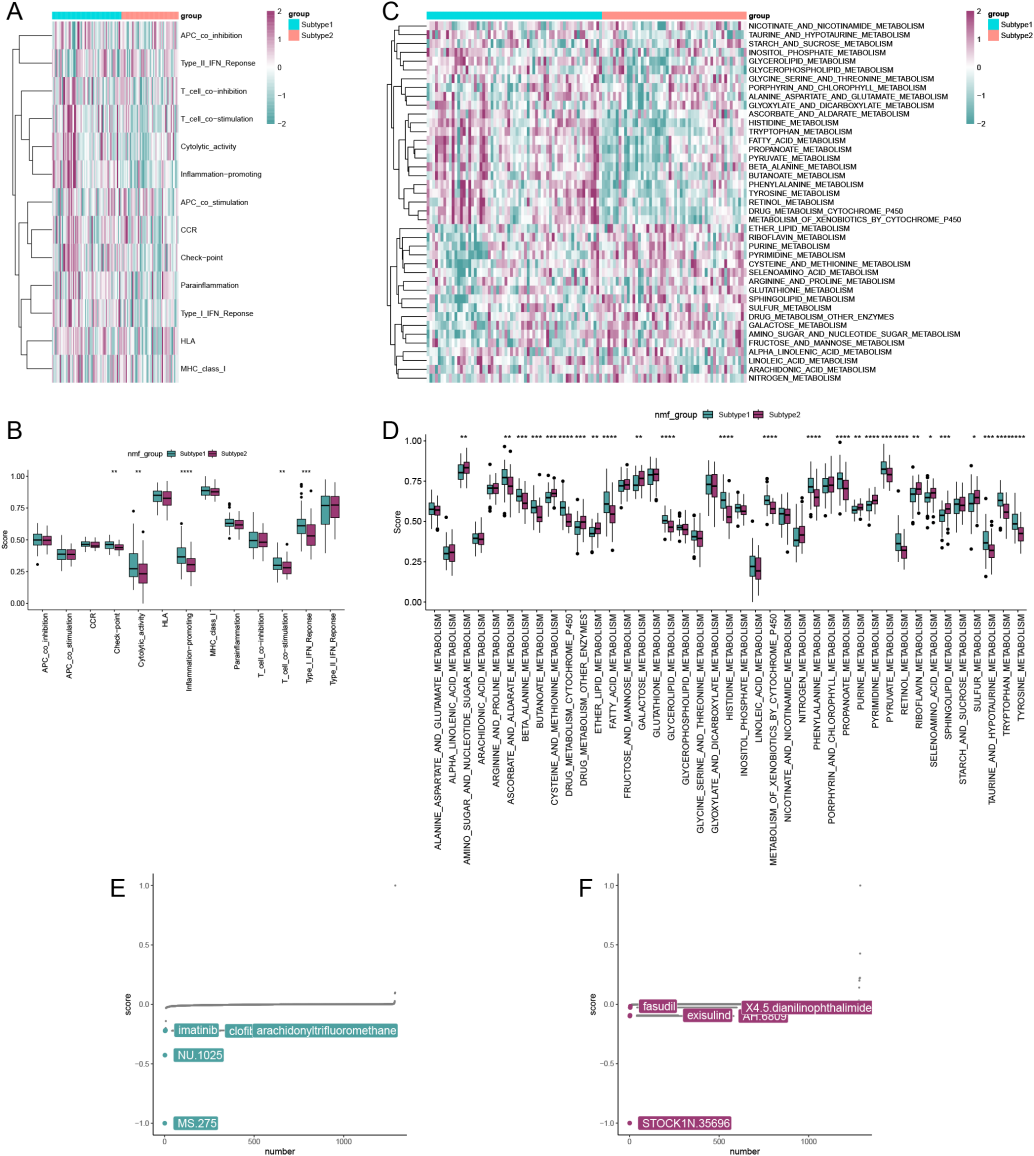
Comparison of activity and drug analysis of the two isoforms. **(A)** Heatmap showing immunoreactivity of samples from both isoforms. **(B)** Rank sum test analyzing the differential expression levels of immune-related pathway activities in the two subtypes. **(C)** Heatmap showing metabolic activity of the two subtype samples. **(D)** Rank sum test analyzing the differential expression levels of metabolism-related pathway activities in the two subtypes. Results of drug analysis available for treatment of subtype 1 patients **(E)** and subtype 2 patients **(F)**. (*p < 0.05, **p < 0.01, ***p < 0.001, ****p < 0.0001).

**Figures 8A, C** present the immune and metabolic pathway activity scores for the two subclusters. **Figure 8B** displays the differential expression levels of immune-related pathway activities between the two subclusters, indicating that subcluster 1 exhibits higher immune activity. Similarly, an analysis of metabolic pathway activities revealed that most metabolic pathways were more active in subcluster 1 (**Figure 8D**). Furthermore, the CMap drug analysis identified the top five drugs for treating patients in subcluster 1 as MS.275, NU.1025, imatinib, clofibrate, and arachidonyltrifluoromethane (**Figure 8E**). In contrast, the top five drugs for treating patients in subcluster 2 included STOCK1N.35696, exisulind, AH.6809, X4.5.dianilinophthalimide, and fasudil (**Figure 8F**). These findings highlight notable differences in immune and metabolic activities between subclusters and suggest potential therapeutic options tailored to each subtype.

### Protein annotation and functional analysis of subtypes

**Figure 9:**
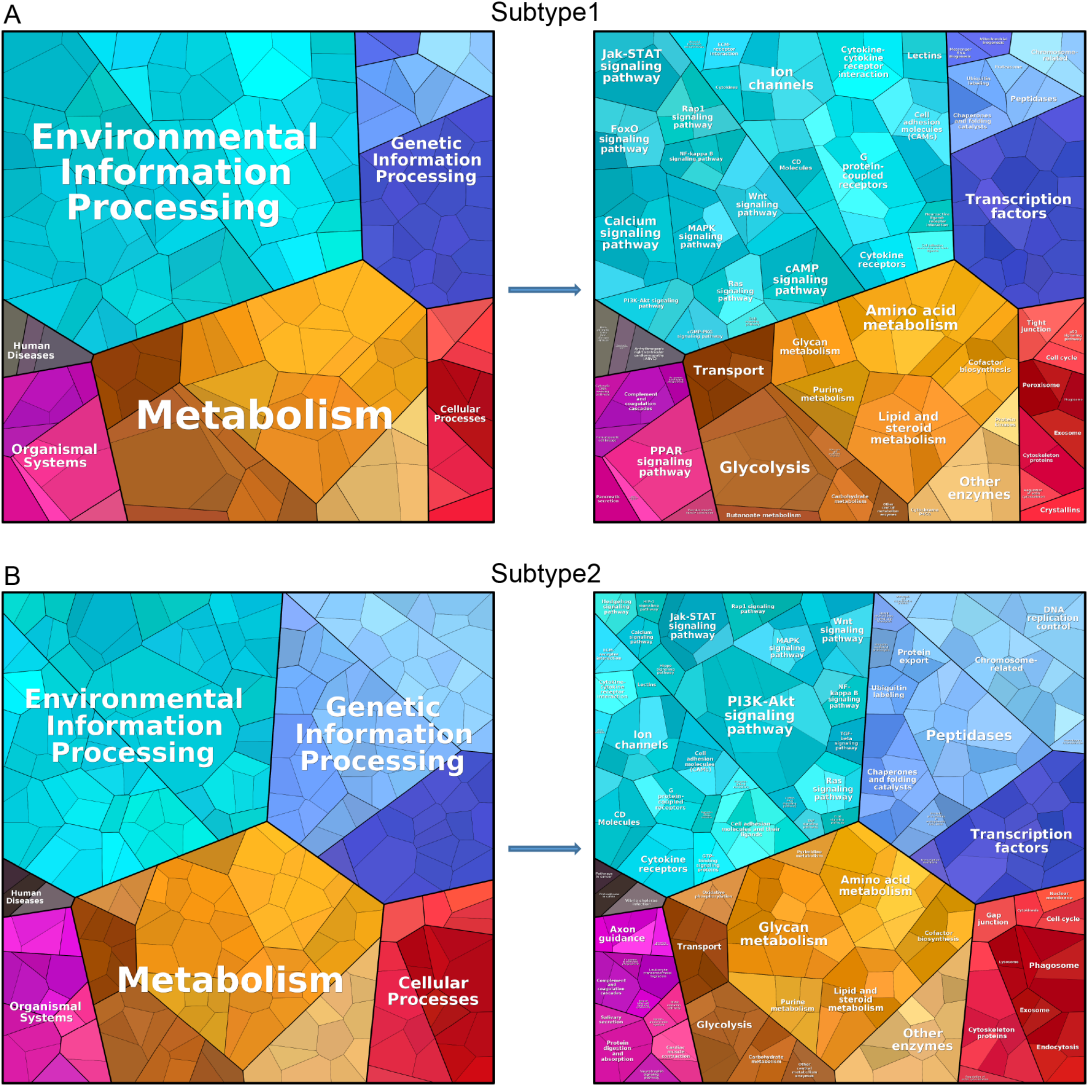
Protein annotation and functional analysis. Protein annotation and functional analysis of isoform 1 **(A)** and isoform 2 **(B)**.

Using the Proteomap database, we conducted protein annotation and functional analysis for both subcluster 1 and subcluster 2. The analysis encompassed categories such as environmental information processing, metabolism, genetic information processing, human diseases, organismal systems, and cellular processes. **Figure 9A** illustrates that subcluster 1 is primarily enriched in environmental information processing and metabolism, with further details provided on specific functions within these six categories. Similarly, **Figure 9B** shows that subcluster 2 is also mainly enriched in environmental information processing and metabolism, along with a detailed breakdown of its specific functions across the six categories.

### GSVA of subtypes

**Figure 10:**
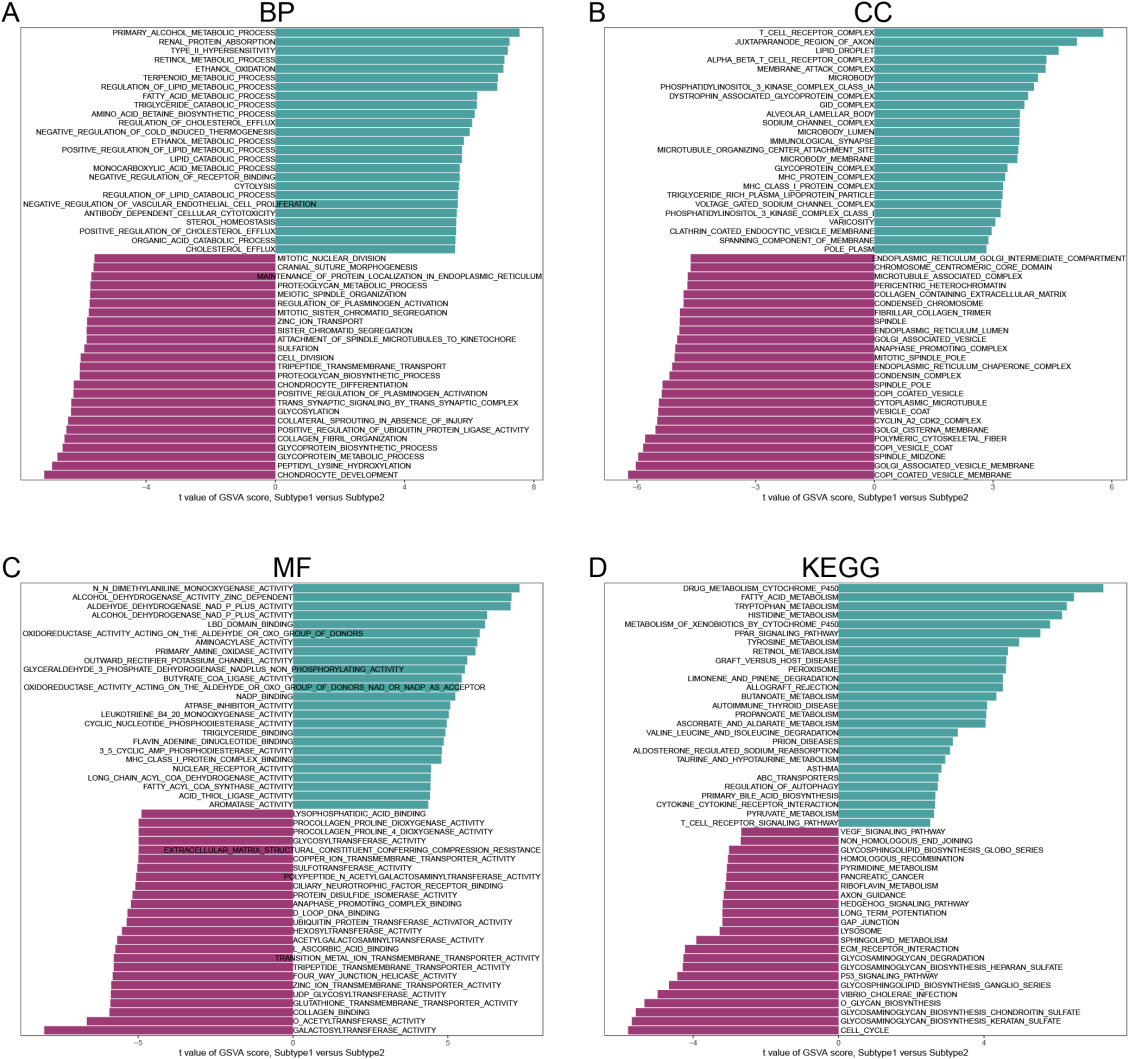
Analysis of gene set variants for subtype 1 and subtype 2. **(A)** BP. **(B)** CC. **(C)** MF. **(D)** KEGG. (Sorted by t-value of GSVA score).

In the BP category, several processes showed significant activity in both subclusters, including Primary Alcohol Metabolic Process, Renal Protein Absorption, Retinol Metabolic Process, Terpenoid Metabolic Process, Chondrocyte Development, and Glycoprotein Metabolic Process (**Figure 10A**). In terms of CC (**Figure 10B**), significant enrichment was observed for components such as the T Cell Receptor Complex, Alpha Beta T Cell Receptor Complex, COPI Coated Vesicle Membrane, and Golgi Associated Vesicle Membrane in both subclusters. For MF (**Figure 10C**), activities such as N, N-Dimethylaniline Monooxygenase Activity, Alcohol Dehydrogenase Activity (Zinc-Dependent), Galactosyltransferase Activity, and O-Acetyltransferase Activity were notably active in both subclusters. Finally, in the KEGG pathway analysis (**Figure 10D**), immune and metabolic-related pathways were significantly activated in both subclusters.

### Co-expression network construction of subtypes

**Figure 11:**
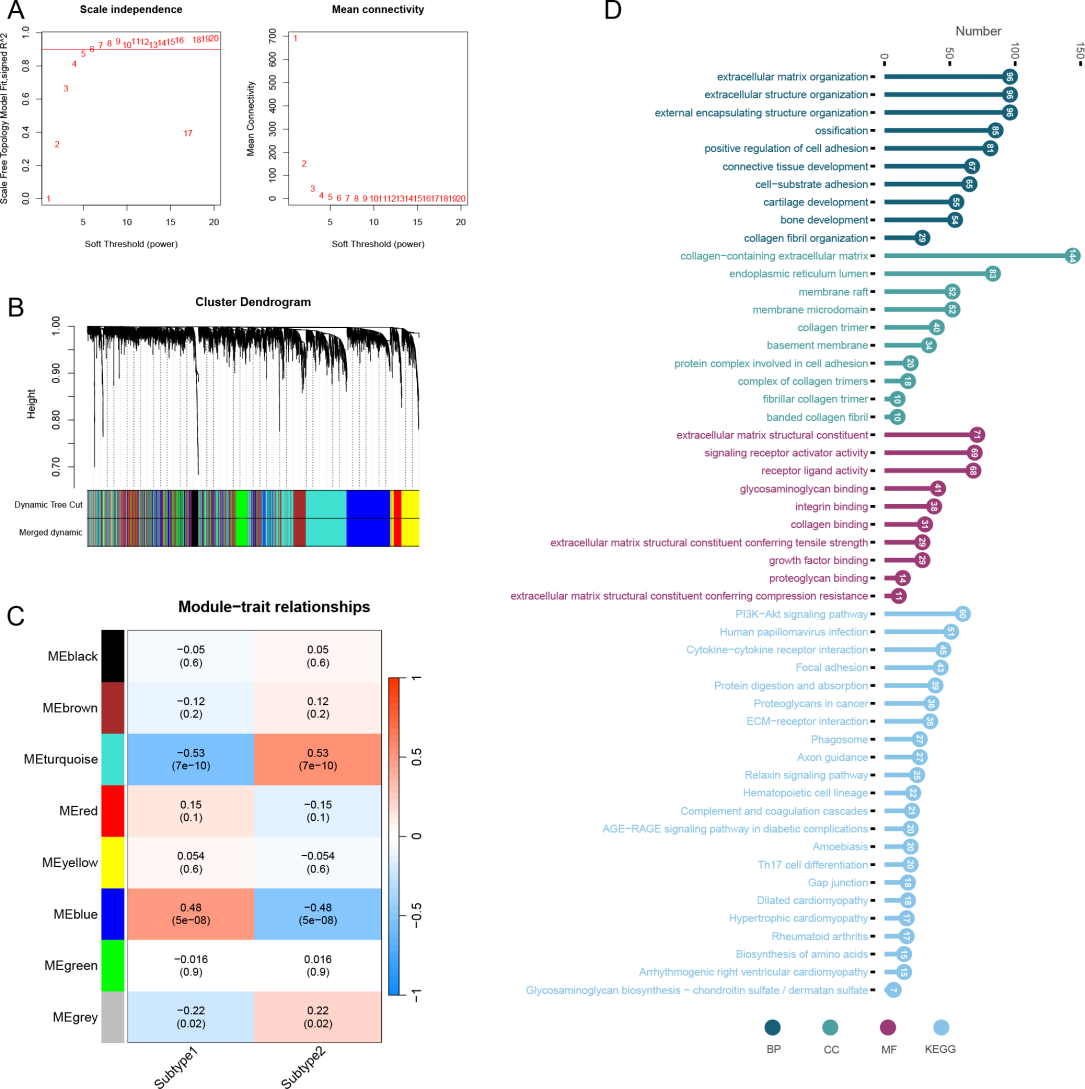
Weighted gene co-expression network analysis of subtypes. **(A)** Optimal soft threshold screening (The vertical axis of the left panel represents the square of the correlation coefficient between log(k) and log(p(k)) in the corresponding network; the vertical axis of the right panel represents the mean of the neighbour-joining function of all genes in the corresponding gene module.). **(B)** Module clustering analysis. **(C)** Correlation analysis of modules and subtypes. **(D)** Enrichment analysis of turquoise module genes including BP, CC, MF and KEGG.

We set the optimal soft threshold to 5, which allowed us to construct a scale-free network (**Figure 11A**). Following this, we merged similar modules together (**Figure 11B**). Through correlation analysis between modules and traits, we discovered that the turquoise module exhibited the strongest correlation with the two subclusters (**Figure 11C**). Furthermore, genes within the turquoise module were significantly enriched in processes such as extracellular matrix organization, extracellular structure organization, and collagen-containing extracellular matrix. Other notable enrichments included endoplasmic reticulum lumen, extracellular matrix structural constituent, signaling receptor activator activity, as well as pathways like the PI3K-Akt signaling pathway, Human papillomavirus infection, and Cytokine-cytokine receptor interaction (**Figure 11D**).

### Single-cell data preprocessing results

**Figure 12:**
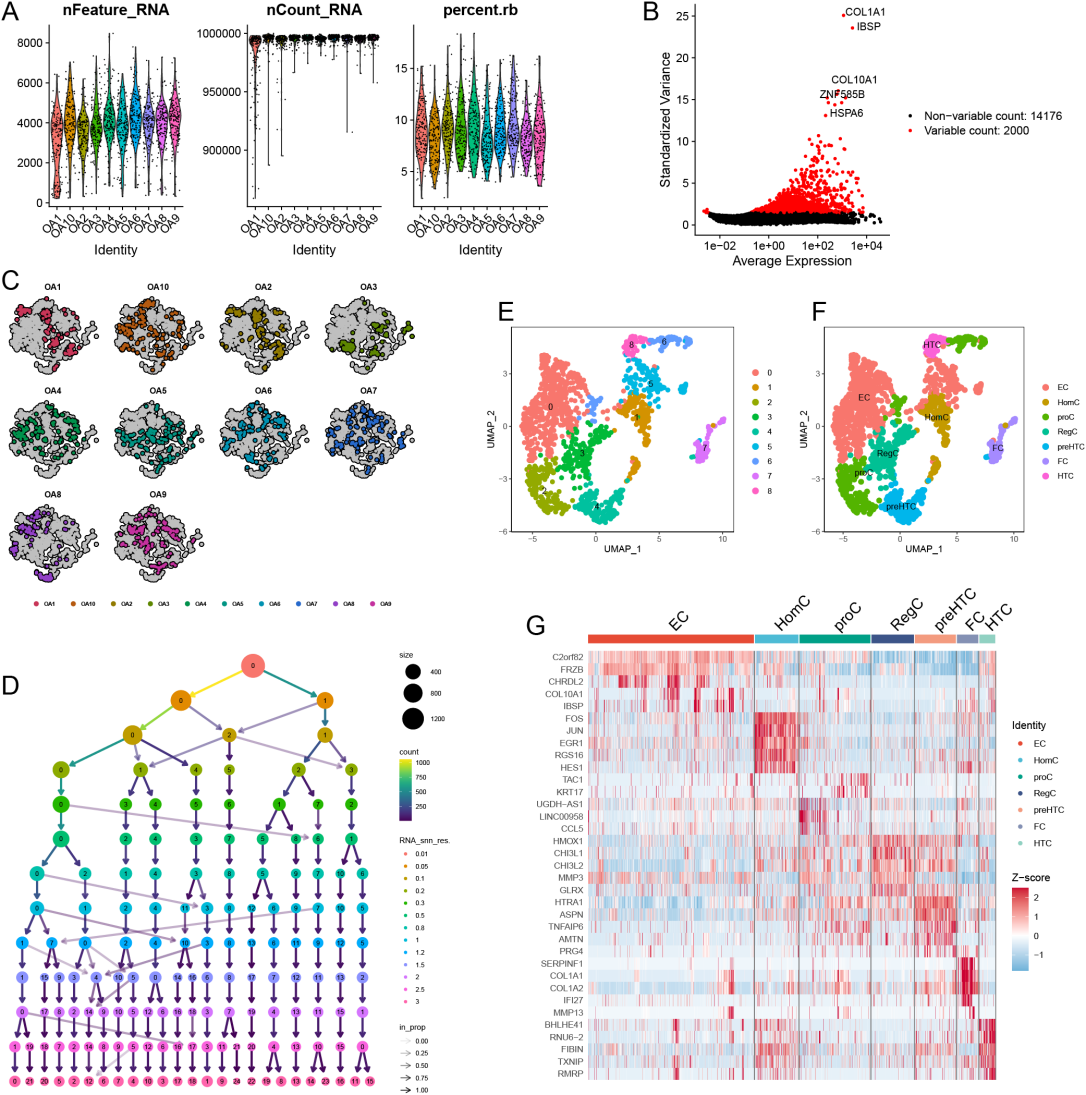
Single-cell data preprocessing. **(A)** The gene counts per cell (nFeature_RNA), number of unique molecular identifiers (UMIs) per cell (nCount_RNA), and percentage of mitochondrial genes per cell (percent.mt) of the single-cell RNA-seq data. **(B)** The variance plot showed genes in all cells, red dots represent the top 2000 highly variable genes. **(C)** Data before dimensionality reduction. **(D)** Dimensionality reduction processing of data. **(E)** UMAP presentation of the downscaling results. **(F)** Cellular annotation of subpopulations. **(G)** Highly variable genes for each subpopulation.

The expression profiles for each sample are displayed in **Figure 12A**. Following this, we identified 2000 highly variable genes (**Figure 12B**). **Figure 12C** illustrates the initial clustering of cell types. By performing dimensionality reduction clustering on the cells (**Figure 12D**), we discovered a total of nine distinct cell subtypes (**Figure 12E**). Integrating these findings with previous research, we characterized the identified subtypes as EC, HomC, proC, RegC, preHTC, FC, and HTC (**Figure 12F**). Finally, the heatmap in **Figure 12G** presents the highly variable genes associated with each subtype, providing insights into the gene expression patterns that distinguish these cellular populations.

### Single-cell expression and pseudotime analysis

**Figure 13:**
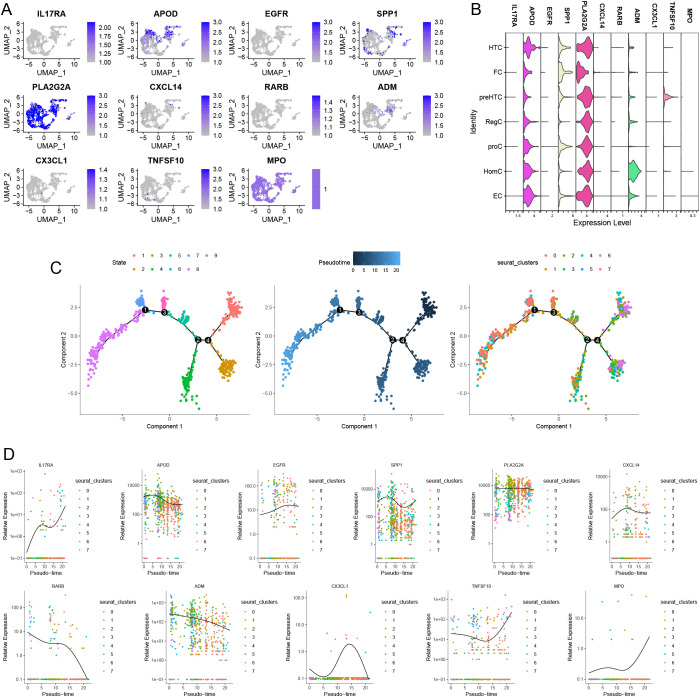
Distribution of Immune-metabolism related hub genes in OA based on single-cell RNA sequencing data. feature **(A)** and violin plots **(B)** showing the distribution of 11 Immune-metabolism related hub genes in various celltypes. **(C)** The 9 states of EC differentiation (left). Differences in the time series of cell differentiation (middle). Dark blue indicates earlier differentiation and light blue indicates later differentiation. All EC were differentiated into 8 clusters (right). **(D)** 11 Immune-metabolism related hub genes produce expression changes in the proposed time series.

**Figures 13A, B** display the distribution and expression of immune-metabolic related hub genes across different cell types. In addition to CX3CR1 and ADIPOQ, we identified the expression of IL17RA, APOD, EGFR, SPP1, PLA2G2A, CXCL14, RARB, ADM, CX3CL1, TNFSF10, and MPO in single cells. Notably, APOD, SPP1, and PLA2G2A exhibited higher expression levels compared to other immune-metabolic related genes across all seven cell types. Given that previous studies indicated high metabolic activity in ECs, we conducted a simulated analysis of the differentiation trajectories of all ECs. As shown in **Figure 13C**, chondrocytes displayed seven distinct states of differentiation, each represented by a different color. We observed that darker shades of blue corresponded to earlier stages of cell differentiation, indicating that EC cells differentiate from left to right over time, with the lightest blue representing the most recently differentiated cells. Throughout the differentiation process, the expression levels of IL17RA, APOD, EGFR, SPP1, PLA2G2A, CXCL14, RARB, ADM, CX3CL1, TNFSF10, and MPO exhibited notable changes (**Figure 13D**).

### Cell communication analysis

**Figure 14:**
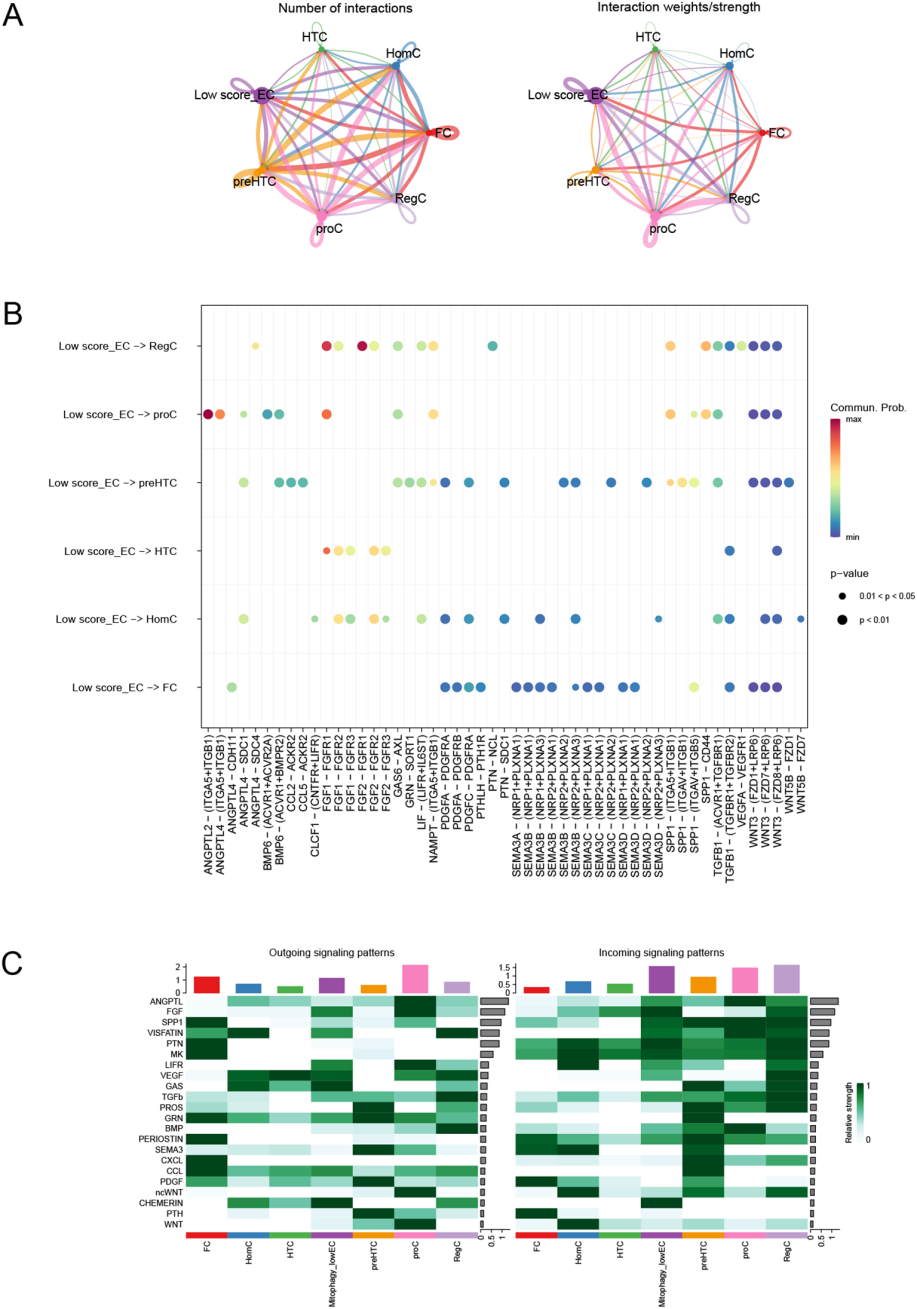
Cell communication analysis. **(A)** Number of interactions or total interaction strength (weights) between cell groups. **(B)** Intercellular communication mediated by ligands and signaling pathways. **(C)** Identification of signals that contribute most to the output and input signals of EC cell taxa.

Through cell communication analysis, we found that low-scoring EC (endothelial) cells are associated with various other cell types (**Figure 14A**). We further examined the ligands and signaling pathways that mediate the interactions between low-scoring EC cells and other cell types (**Figure 14B**). Ultimately, our analysis revealed that the signal contributing most significantly to both the output and input for the EC cell population is CHEMEPIN (**Figure 14C**).

### GWAS analysis

By analyzing GWAS data, we identified pathogenic regions associated with single nucleotide polymorphisms (SNPs) for 13 immune-metabolic related hub genes (**Supplementary Figures 3A-M**). Additionally, by examining the chromosomal location information of these genes, we further elucidated their genetic context (**Supplementary Figure 3N**).

### RT-PCR validation results

**Figure 15:**
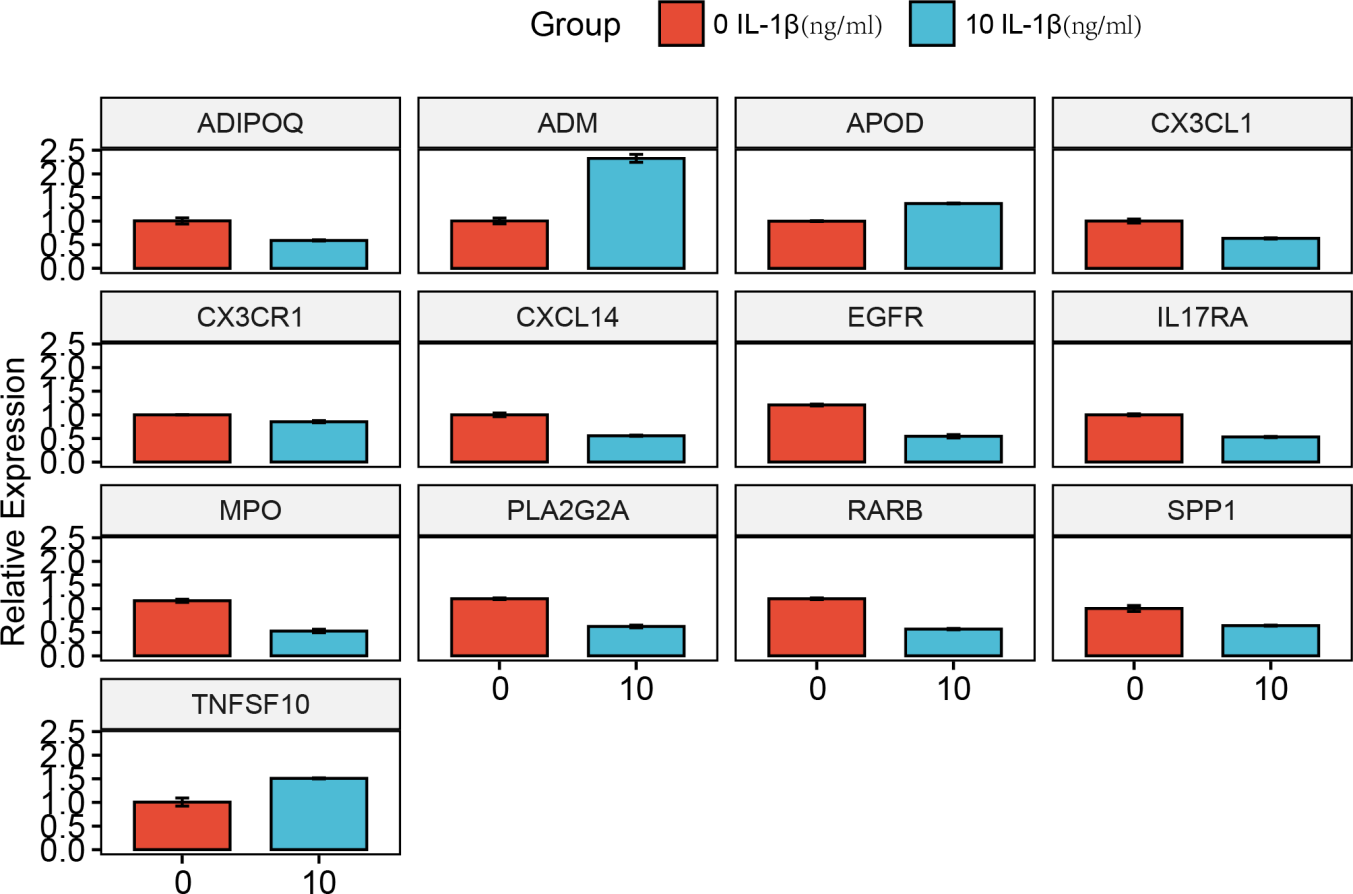
Experimental validation of key gene expression. Relative mRNA expression of ADIPOQ, ADM, APOD, CX3CL1, CX3CR1, CXCL14, EGFR, IL17RA, MPO, PLA2G2A, RARB, SPP1, and TNFSF10.

Compared with the0 IL-β(ng/ml) group, the 10 IL-β(ng/ml) group exhibited higher expression levels of the genes ADIPOQ, CX3CL1, CX3CR1, CXCL14, EGFR, IL17RA, MPO, PLA2G2A, RARB, and SPP1. Conversely, the 10 IL-β(ng/ml) group showed lower expression levels of the genes ADM, APOD, and TNFSF10 than the 0 IL-β(ng/ml) group (**Figure 15**).

## Discussion

Despite being a prevalent cause of pain and disability in humans, OA remains inadequately treated for many patients. Historically, the therapeutic focus has primarily centered on alleviating clinical symptoms. However, early diagnosis and prompt intervention are crucial for improving the prognosis of individuals with osteoarthritis. Consequently, there is an urgent need to identify biomarkers that can facilitate the recognition and treatment of OA.

An increasing body of research indicates a close relationship between immune responses, metabolism, and the pathogenesis of OA. In this study, we employed bioinformatics approaches to identify immune-metabolic related genes and pinpointed those exhibiting expression changes in OA. Notably, the gene set composed of these genes was significantly activated in the context of OA. GO and KEGG analyses revealed that these genes were notably enriched in processes such as the regulation of inflammatory response, positive regulation of cytokine production, regulation of lipid metabolic processes, the PI3K-Akt signaling pathway, cytokine-cytokine receptor interactions, and Ras signaling pathway. Initially regarded as “wear-and-tear” arthritis, OA is now understood to involve inflammatory mediators released by cartilage, bone, and synovium. Recent evidence suggests that inflammatory mechanisms associated with OA include innate immunity, metabolic syndrome, and low-grade inflammation induced by inflammaging (13). Inflammation is a variable hallmark of OA, correlating with joint symptoms and disease progression (14). The elevation of systemic and local inflammatory cytokines and senescence-associated molecules promotes cartilage degradation, while antigens from damaged joints further trigger inflammation through inflammasome activation (15). Pro-inflammatory cytokines serve as critical mediators of the metabolic disturbances and enhanced catabolism observed in OA-related articular tissues. Currently, IL-1β, TNF, and IL-6 are recognized as the principal pro-inflammatory cytokines involved in the pathogenesis of OA; additionally, other factors such as IL-15, IL-17, IL-18, IL-21, leukemia inhibitory factor (LIF), and various chemokines have also been implicated in the disease’s onset (16). Moreover, lipids, including phospholipids and fatty acids along with their derivatives, have been associated with the inflammatory processes in OA (17). Dysregulation of extracellular matrix metabolism, lipid metabolic disorders, and upregulation of the senescence-associated secretory phenotype are all mechanisms linked to the pathogenesis of OA (18). In the synovial tissue, synovial fluid, and peripheral blood of individuals with OA, activated macrophages are regulated by mTOR, NF-κB, JNK, PI3K/Akt, and other signaling pathways, differentiating into M1 or M2 subtypes. The activation state of macrophages and the M1/M2 ratio are closely related to the severity of OA (19). The RAS pathway participates in several signaling cascades, including NF-κB, JNK, VEGFR/Tie-2, and Axna2/Axna2R, which may represent potential therapeutic targets for OA (20). Through ssGSEA pathway analysis, we found significant activation of immune and metabolic pathways in OA. Additionally, WGCNA identified multiple immune and metabolic biological processes closely associated with OA, including monocarboxylic acid metabolic process, regulation of lipid metabolic process, organic hydroxy compound metabolic process, generation of precursor metabolites and energy, lipid and atherosclerosis, fatty acid biosynthesis, and glycine, serine, and threonine metabolism. These findings further substantiate the reliability of our results.

In this study, we identified 13 immune-metabolic hub genes associated with OA, namely CX3CR1, ADIPOQ, IL17RA, APOD, EGFR, SPP1, PLA2G2A, CXCL14, RARB, ADM, CX3CL1, TNFSF10, and MPO. Prior research has indicated a potential association between the rs182052 polymorphism in the ADIPOQ gene and the risk of knee OA (21). Subsequent studies have demonstrated that the rs1501299 polymorphism within the ADIPOQ gene increases the risk of knee OA (22). The anti-apoptotic peptide ADM promotes apoptosis in inflammatory arthritis synovial cells and dedifferentiation of chondrocytes by enhancing oxidative stress and the production of pro-inflammatory cytokines (23, 24). CX3CL1, a member of the CX3C chemokine family, has been shown to enhance the production of matrix metalloproteinase-3 (MMP-3) in OA fibroblasts through the activation of the CX3CR1, c-Raf, MEK, ERK, and NF-κB signaling pathways (25). MMP-3 is involved in the processes that contribute to OA pathogenesis through its role in matrix degradation (26). In temporomandibular joint OA, chondrocyte apoptosis, mediated by the activation of the p38-CX3CL1 pathway, enhances the chemotactic effect of osteoclast precursors towards osteoblasts, thereby promoting local osteoclast activation (27). The CX3CL1 receptor, CX3CR1, can promote the proliferation and apoptosis of OA chondrocytes via the Wnt/β-catenin signaling pathway (28). Research has indicated that the absence of epidermal growth factor receptor (EGFR) specifically in cartilage accelerates the onset of knee OA (29, 30). Inhibition of EGFR ubiquitination can suppress extracellular matrix degradation while activating chondrocyte autophagy, thus serving a protective role against OA progression (31). EGFR signaling is essential for maintaining the number and characteristics of superficial chondrocytes, promoting the expression of proteoglycan 4 (Prg4), and stimulating the lubricating function of cartilage surfaces. Furthermore, defects in EGFR significantly disrupt the arrangement of collagen fibers within joint cartilage and markedly reduce the surface modulus of cartilage (32). In an OA mouse model, inhibition of IL-6 through IL-17RA-mediated pathways was found to suppress synovitis (33). The upregulation of IL-17RA expression in cartilage and synovium during the later stages of OA suggests its critical role in the pathophysiology of the disease (34). Numerous studies have reported elevated expression levels of SPP1 in both OA cartilage and synovium (35). MicroRNA-186 has been shown to inhibit chondrocyte apoptosis in OA mouse models by repressing the activation of the PI3K-AKT pathway via SPP1 (36). Overexpression of TNFSF10 may promote proliferation and inflammation while inhibiting apoptosis, thereby facilitating OA progression through regulation of the miR-376-3p/FGFR1 axis (37). As an immune-related biomarker, APOD exhibits high diagnostic efficacy for OA (38). Additionally, increased expression of PLA2G2A in the cartilage of OA patients indicates its degradative effects on cartilage and suggests it may serve as a potential therapeutic target for OA (39). Elevated expression of MPO in the OA synovium could also be utilized as an early diagnostic marker for the condition (38). These findings robustly support our results, indicating that the expression levels of CX3CR1, ADIPOQ, IL17RA, APOD, EGFR, SPP1, PLA2G2A, CXCL14, RARB, ADM, CX3CL1, TNFSF10, and MPO are significantly correlated with the mechanisms underlying OA pathogenesis.

In the progression of OA, metabolic regulation plays a crucial role in maintaining cartilage functionality and self-repair mechanisms. Abrupt changes in metabolic regulation can lead to functional abnormalities, such as impaired extracellular matrix synthesis. Moreover, researchers have identified a newly discovered cell type known as ECs, characterized by elevated metabolic rates. These cells are closely associated with processes involving the tricarboxylic acid cycle, glycolysis, oxidative phosphorylation, and lipid and amino acid metabolism, suggesting that effector chondrocytes exhibit enhanced activity in energy metabolism. Additionally, regulatory chondrocytes express high levels of specific markers associated with the innate immune system, indicating that these cells may possess functions related to immune cell activation. These novel insights into chondrocyte functionality deepen our understanding of OA. Through high-resolution single-cell transcriptomic sequencing of samples from OA patients, researchers have revealed the presence of distinct functional chondrocyte subtypes within human osteoarthritic cartilage (40). Utilizing bioinformatics algorithms, we elucidated the spatial distribution patterns of these cell subtypes within cartilage tissue, as well as their temporal distribution patterns throughout the progression of OA. Seven distinct chondrocyte subtypes were identified, revealing the expression distribution patterns of hub immune-metabolic-related genes while also analyzing the pseudotemporal variation characteristics of effector chondrocytes. Pseudotime trajectory analysis demonstrated that these hub immune-metabolic-related genes are involved in the transitions between chondrocyte subpopulations, underscoring the significant role of immune metabolism in the development of OA.

Of course, our study has some limitations. First, although we use publicly available data for analysis, the reliability of the data may be a potential problem. But we have worked hard to ensure data quality and consistency. The use of independent datasets or experimental validation will further ensure the reliability of our findings. Second, although our prediction model shows promising results, it needs to be clinically validated in the future. Finally, as this study focuses on bioinformatics analysis, future experimental studies should aim to clarify the biological relevance of these genes, explore their interactions in cellular processes, and investigate potential therapeutic targets.

In summary, the interplay between immune metabolism and the pathogenesis of OA is closely intertwined. Our study not only offers a comprehensive molecular understanding of the immune-metabolic characteristics associated with OA but also identifies potential biomarkers and therapeutic targets for future treatment strategies. These findings may contribute to the development of innovative therapeutic approaches aimed at enhancing the prognosis and quality of life for OA patients.

## Data Availability

The original contributions presented in the study are included in the article/**Supplementary Material**. Further inquiries can be directed to the corresponding authors.

## Supplementary material

Supplementary Figure 1 Heatmap of gene correlations.

Supplementary Figure 2 Intersection genes identified by machine learning.

Supplementary Figure 3 GWAS analysis of chromosomal results for ADIPOQ **(A)**, ADM **(B)**, APOD **(C)**, CX3CL1 **(D)**, CX3CR1 **(E)**, CXCL14 **(F)**, EGFR **(G)**, IL17RA **(H)**, MPO **(I)**, PLA2G2A **(J)**, RARB **(K)**, SPP1 **(L)** and TNFSF10 **(M)** region. **(N)** Chromosomal locus information.
