# Supplementary figures and images for: Integrated multi-omics and machine learning reveals immune-metabolic signatures in osteoarthritis: from bulk RNA-seq to single-cell resolution
Source: Front Immunol. 2025 Jun 16;16:1599930. doi: 10.3389/fimmu.2025.1599930 (PMC12206867; doi:10.3389/fimmu.2025.1599930)

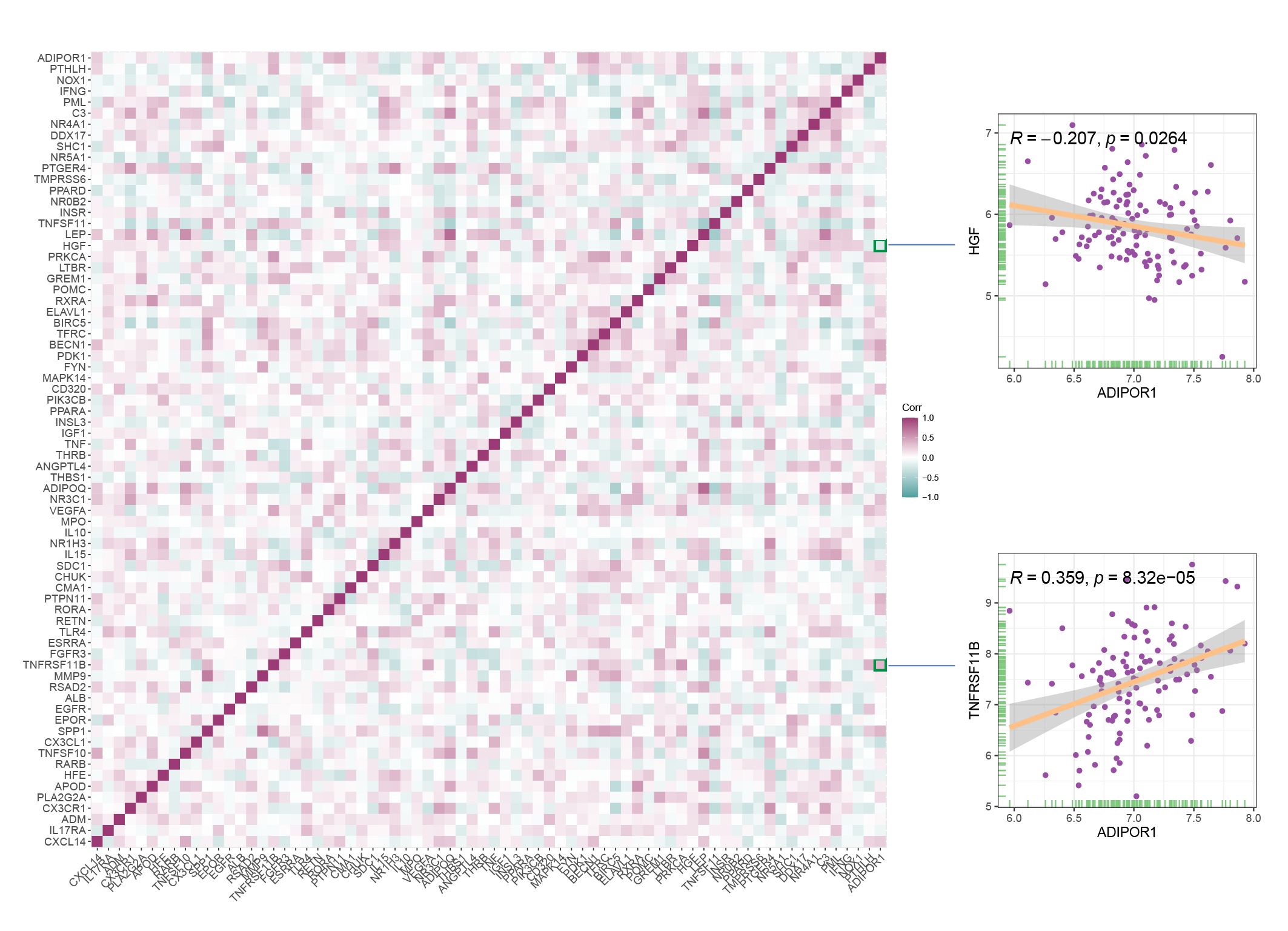

Supplement: Supplementary Figure 1 — Heatmap of gene correlations. [file Image1.tif]

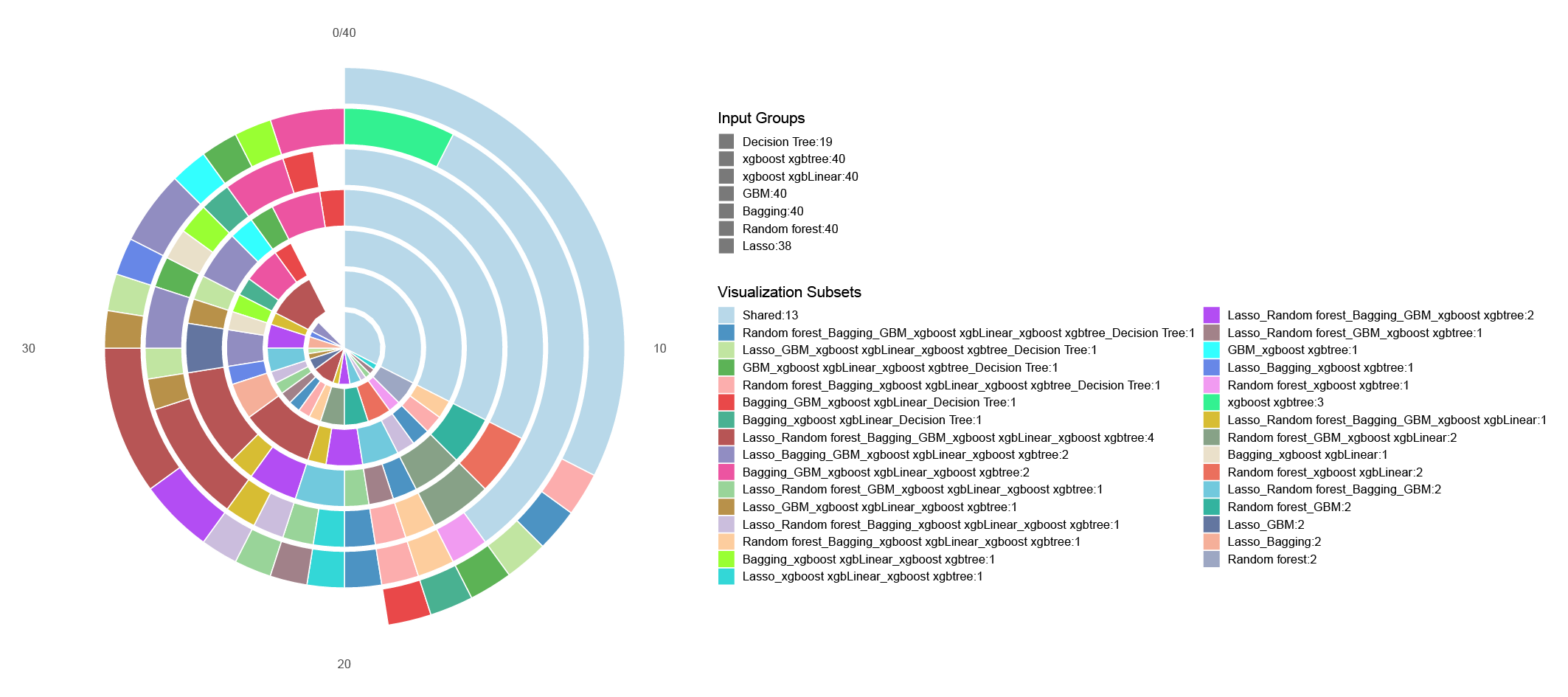

Supplement: Supplementary Figure 2 — Intersection genes identified by machine learning. [file Image2.tif]

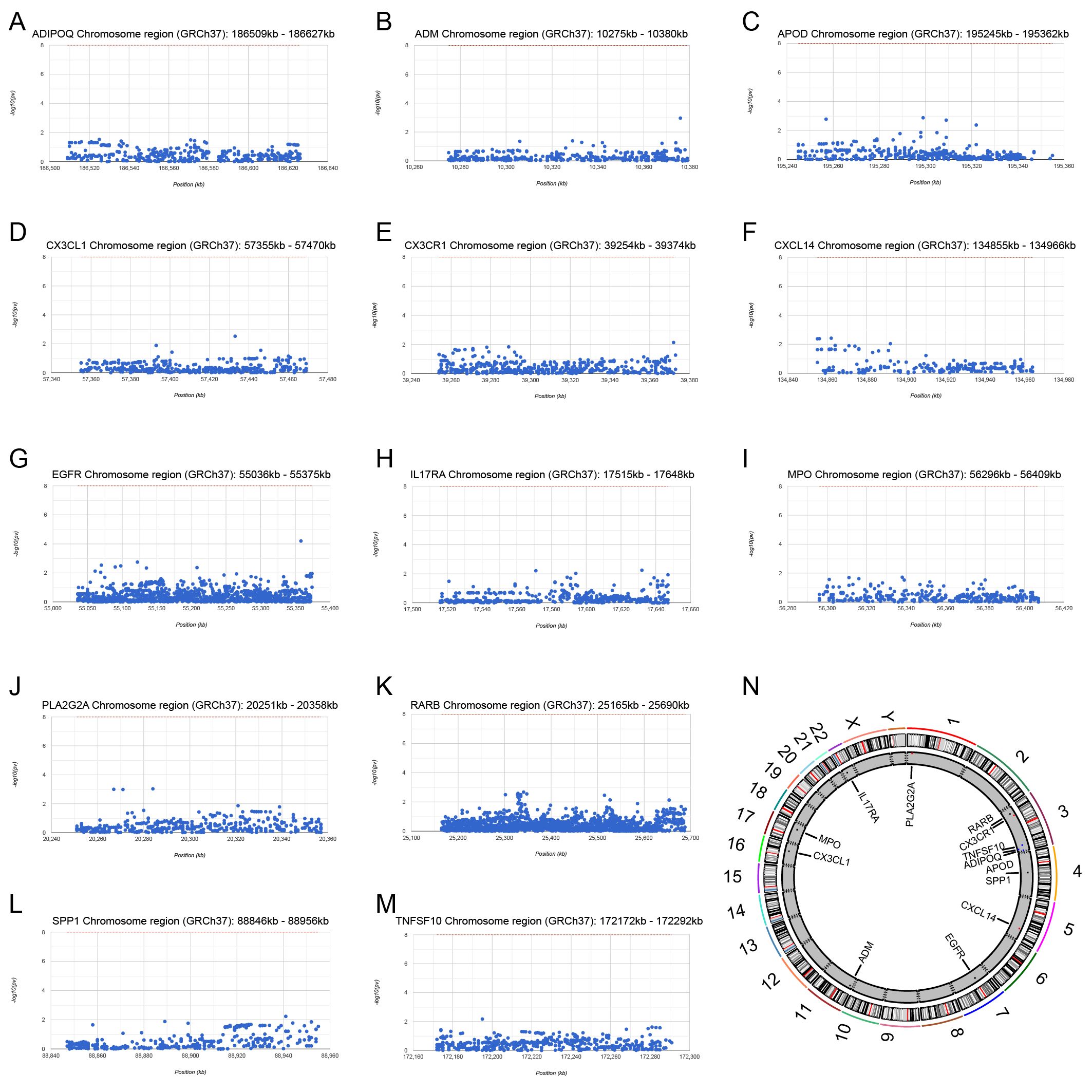

Supplement: Supplementary Figure 3 — GWAS analysis of chromosomal results for ADIPOQ (A), ADM (B), APOD (C), CX3CL1 (D), CX3CR1 (E), CXCL14 (F), EGFR (G), IL17RA (H), MPO (I), PLA2G2A (J), RARB (K), SPP1 (L) and TNFSF10 (M) region. (N) Chromosomal locus information. [file Image3.tif]
