# Supplementary material for: Integrated multi-omics and machine learning reveals immune-metabolic signatures in osteoarthritis: from bulk RNA-seq to single-cell resolution
Source: Front Immunol. 2025 Jun 16;16:1599930. doi: 10.3389/fimmu.2025.1599930 (PMC12206867; doi:10.3389/fimmu.2025.1599930)
Supplement: Supplementary file 4 [file Table1.docx]

**Supplementary table 1. Details of the datasets.**

| Dataset | GPL Platform | Sample | Year | Data link |
| --- | --- | --- | --- | --- |
| GSE117999 | GPL20844 | 12 OA samples and 12 control samples | 2018 | https://www.ncbi.nlm.nih.gov/geo/query/acc.cgi?acc=GSE117999 |
| GSE51588 | GPL13497 | 40 OA samples and 10 control samples | 2018 | https://www.ncbi.nlm.nih.gov/geo/query/acc.cgi?acc=GSE51588 |
| GSE55235 | GPL96 | 10 OA samples and 10 control samples | 2018 | https://www.ncbi.nlm.nih.gov/geo/query/acc.cgi?acc=GSE55235 |
| GSE55457 | GPL96 | 10 OA samples and 10 control samples | 2018 | https://www.ncbi.nlm.nih.gov/geo/query/acc.cgi?acc=GSE55457 |
| GSE57218 | GPL6947 | 33 OA samples and 7 control samples | 2018 | https://www.ncbi.nlm.nih.gov/geo/query/acc.cgi?acc=GSE57218 |
| GSE82107 | GPL570 | 10 OA samples and 7 control samples | 2019 | https://www.ncbi.nlm.nih.gov/geo/query/acc.cgi?acc=GSE82107 |
| GSE98918 | GPL20844 | 12 OA samples and 12 control samples | 2021 | https://www.ncbi.nlm.nih.gov/geo/query/acc.cgi?acc=GSE98918 |
| GSE12021 | GPL97 | 10 OA samples | 2018 | https://www.ncbi.nlm.nih.gov/geo/query/acc.cgi?acc=GSE12021 |
